# Combination of induced pluripotent stem cell-derived motor neuron progenitor cells with irradiated brain-derived neurotrophic factor over-expressing engineered mesenchymal stem cells enhanced restoration of axonal regeneration in a chronic spinal cord injury rat model

**DOI:** 10.1186/s13287-024-03770-9

**Published:** 2024-06-18

**Authors:** Jang-Woon Kim, Juryun Kim, Soon Min Lee, Yeri Alice Rim, Young Chul Sung, Yoojun Nam, Hyo-Jin Kim, Hyewon Kim, Se In Jung, Jooyoung Lim, Ji Hyeon Ju

**Affiliations:** 1 CiSTEM laboratory, Catholic iPSC Research Center (CiRC), College of Medicine, The Catholic University of Korea, Seoul, 137-701 Republic of Korea; 2 Department of Biomedicine & Health Science, Seoul St. Mary’s Hospital, College of Medicine, The Catholic University of Korea, Seoul, Republic of Korea; 3 Division of Rheumatology, Department of Internal Medicine, Seoul St. Mary’s Hospital, Institute of Medical Science, College of Medicine, The Catholic University of Korea, Seoul, 137-701 Republic of Korea; 4 YiPSCELL, Inc., Seoul, Republic of Korea; 5 SL BiGen, Inc., Incheon, Republic of Korea

**Keywords:** BDNF over-expressing engineered mesenchymal stem cell, Induced pluripotent stem cell-derived motor neuron progenitor cell, Chronic spinal cord injury, Combination cell transplantation

## Abstract

**Background:**

Spinal cord injury (SCI) is a disease that causes permanent impairment of motor, sensory, and autonomic nervous system functions. Stem cell transplantation for neuron regeneration is a promising strategic treatment for SCI. However, selecting stem cell sources and cell transplantation based on experimental evidence is required. Therefore, this study aimed to investigate the efficacy of combination cell transplantation using the brain-derived neurotrophic factor (BDNF) over-expressing engineered mesenchymal stem cell (BDNF-eMSC) and induced pluripotent stem cell-derived motor neuron progenitor cell (iMNP) in a chronic SCI rat model.

**Method:**

A contusive chronic SCI was induced in Sprague-Dawley rats. At 6 weeks post-injury, BDNF-eMSC and iMNP were transplanted into the lesion site via the intralesional route. At 12 weeks post-injury, differentiation and growth factors were evaluated through immunofluorescence staining and western blot analysis. Motor neuron differentiation and neurite outgrowth were evaluated by co-culturing BDNF-eMSC and iMNP in vitro in 2-dimensional and 3-dimensional.

**Results:**

Combination cell transplantation in the chronic SCI model improved behavioral recovery more than single-cell transplantation. Additionally, combination cell transplantation enhanced mature motor neuron differentiation and axonal regeneration at the injured spinal cord. Both BDNF-eMSC and iMNP played a critical role in neurite outgrowth and motor neuron maturation via BDNF expression.

**Conclusions:**

Our results suggest that the combined transplantation of BDNF- eMSC and iMNP in chronic SCI results in a significant clinical recovery. The transplanted iMNP cells predominantly differentiated into mature motor neurons. Additionally, BDNF-eMSC exerts a paracrine effect on neuron regeneration through BDNF expression in the injured spinal cord.

**Graphical Abstract:**

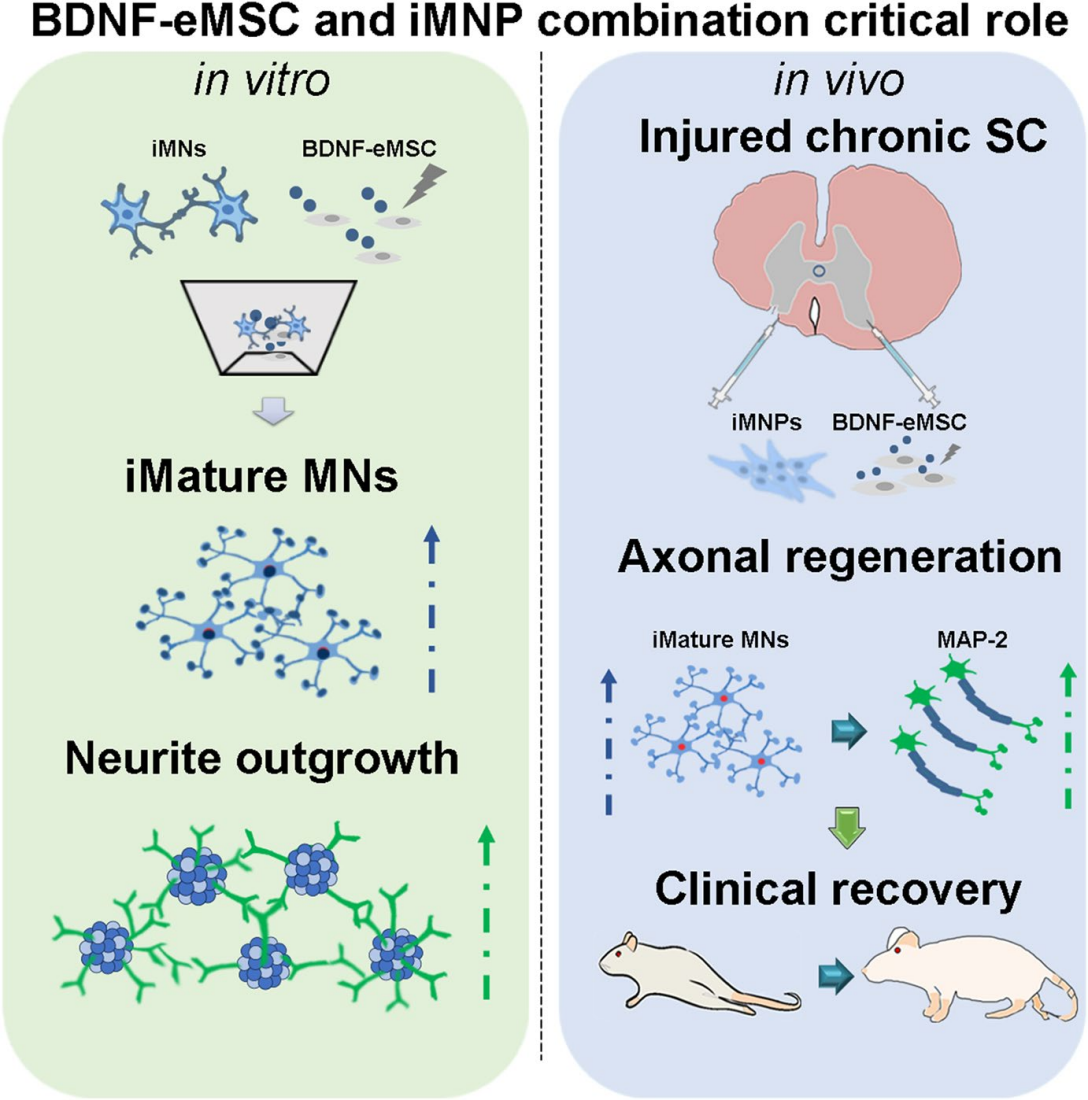

**Supplementary Information:**

The online version contains supplementary material available at 10.1186/s13287-024-03770-9.

## Background

Spinal cord injury (SCI) is a disease that causes motor, sensory, and autonomic dysfunction. It is characterized by various symptoms, including post-injury paralysis, paresthesia, spastic pain, and cardiovascular, bladder, or sexual dysfunction. Severe SCI is a leading cause of death owing to severe autonomic dysfunction and neurogenic shock [1]. Based on a retrospective population-based study conducted between 2011 and 2020, 1,303 traumatic SCI (TSCI) accidents occurred among 4.9 million residents. The recent increase in TSCI incidence has increased its recognition as a global health priority [2]. The causes of TSCI are motor vehicle accidents, falls, work-related injuries, violent crimes, and sport-related injuries [3]. Patients with TSCI experience substantial mortality and morbidity rates, as well as an economic burden, owing to the high cost and complexity of medical care and lost productivity [2].

The pathophysiology of TSCI comprises two phases: primary and secondary. In most clinical situations, the focus is to prevent secondary injury mechanisms that occur following the primary injury. The secondary injury process is divided into acute, sub-acute, and chronic phases based on the time after the injury [3]. After the primary injury, pathological changes, such as chronic inflammation, cell dysfunction, and vascular changes, occur in the injured spinal cord (SC) tissue. These changes activate resident astrocytes, microglia, fibroblasts, and other glial cells at the lesion site and contribute to the infiltration of peripheral immune cells. The interactions between these cells at the lesion site are the basis of glial scarring, which inhibits axonal regeneration and myelination formation [4–7].

Currently, effective treatments for acute and chronic SCI do not exist. Stem cell transplantation has emerged as a promising strategy to inhibit glial scarring and reduce inflammation. Various cell sources are being explored in stem cell transplantation studies for SCI. The transplantation of Schwann cells, neural stem or progenitor cells, olfactory ensheathing cells (OECs), oligodendrocyte precursor cells, and mesenchymal stem cells (MSC) have been investigated as potential therapies for SCI. Stem cell transplantation can be derived from adult and embryonic stem cells (ESC) and induced pluripotent stem cells (iPSC) via direct conversion technology [8–13]. A combined cell transplantation approach is required to treat SCI more effectively because cellular response factors within the injured tissue determine SCI progression [14, 15].

We aimed to confirm the feasibility of a combination cell transplantation strategy in a chronic SCI model. Initially, we used MSC as the first combination cell source. Prior research has reported significant clinical improvement in chronic SCI following MSC transplantation. Astrocytic differentiation of the transplanted cells was predominant at the lesion site. The potential of transplanted cells in chronic SCI has been confirmed; however, further research is required to improve their migration and differentiation into functional cells [16]. Brain-derived neurotrophic factor (BDNF) plays an essential role in neuronal maturation, differentiation, and survival of newly generated neurons via BDNF and TrkB signaling. BDNF has a promising potential as a treatment for central nervous system diseases such as brain disease and SCI; however, its application for neurological diseases is limited [17–19]. BDNF has a short half-life in vivo and cannot cross the blood-brain barrier, posing complex challenges in its application. To overcome these limitations, BDNF overexpression in MSCs has been attempted as a treatment for neurological diseases. Based on previous research, we have engineered human MSCs (hMSCs) to overexpress BDNF. Moreover, previous studies have confirmed that BDNF over-expressing engineered MSC (BDNF-eMSC) after irradiation can enhance their efficacy in facilitating recovery from brain diseases in rodent models [17–19].

Furthermore, previous studies have reported that transplanting ESC-derived and iPSC-derived-motor neuron progenitor cells increased neuronal survival and promoted neurite branching, resulting in functional recovery in SCI models. Recent experimental studies have suggested motor neuron and motor neuron progenitor cells as potential stem cell therapy strategies for SCI [20–23]. We aimed to transplant iPSC-derived motor neuron progenitor cells (iMNP) as a second combination cell source to increase the motor neuron differentiation rate at the lesion site in a chronic SCI model.

Trials of ideal cell types and cell transplantation strategies for chronic SCI are required to achieve effective stem cell transplantation. In this study, we used BDNF-eMSC and iMNP combination cell transplantation in a chronic SCI model. We hypothesized that transplanting BDNF-eMSC and iMNP cells in the severe stage of chronic SCI would induce functional recovery through BDNF expression, mature motor neuron differentiation, and axonal regeneration.

## Materials and methods

### In vitro assay

#### BDNF-eMSC preparation

BDNF-eMSC was established based on the previously reported protocol [17–19, 24, 25] and provided as irradiated form by SL BIGEN, Inc., (Incheno, Korea). Human bone marrow-derived MSCs were purchased from the Catholic Institute of Cell Therapy, South Korea. MSC was cultured in low glucose-containing Dulbecco’s Modified Eagle Medium (DMEM) (Gibco, Grand Island, NY, USA) supplemented with 20% fetal bovine serum (FBS) (Gibco) and 5 ng/mL basic fibroblast growth factor (bFGF) (PeproTech, Rocky Hill, NJ, USA) and BDNF-eMSC was cultured in low glucose-DMEM supplemented with 10% FBS, 10 ng/mL bFGF in a humidified atmosphere of 5% CO_2_ at 37℃.

#### iPSC derived motor neuron progenitor cell generation

Human iPSCs were generated from cord blood mononuclear cells (CBMCs) as previously described using Cyto Tune-iPSC Sendai Reprogramming kit containing Yamanaka factors (A16518, Thermo Fisher Scientific). CBMCs were directly obtained from the Cord Blood Bank of the Seoul St. Mary’s Hospital [26, 27]. The CBMC-derived iPSCs were cultured and maintained in vitronectin-coated plate dishes using Essential 8™ Basal medium (Thermo Fisher Scientific) and supplements (Thermo Fisher Scientific). The differentiation of iPSC into motor neuron using small molecules was performed based on a previously reported protocol [28, 29]. During motor neuron differentiation, we used motor neuron induction medium, including DMEM/F12, Neurobasal medium at 1:1, 1% N_2_, 1% B27, (Thermo Fisher Scientific), 0.1 mM ascorbic acid (Sigma-Aldrich, St Louis, MO, USA), 1X Glutamax, and 1X penicillin/streptomycin (Thermo Fisher Scientific). Induced pluripotent stem cell-derived neuron epithelial progenitor (iNEP) differentiation was induced in a motor neuron induction medium containing CHIR99021 (3 µM, Tocris, Bristol, United Kingdom), 2 µM dorsomorphin homolog 1 (Tocris), and 2 µM SB431542 (Stemgent, Cambridge, MA, USA). The culture medium was changed every other day for 6 days. During the induction of iMNP differentiation, retinoic acid (RA; 0.1 µM, Stemgent) and pumorphamine (Pur; 0.5 µM, Stemgent) were added to the iNEP cells along with 1 µM CHIR99021 (Tocris), 2 µM DMH1 (Tocris), and 2 µM SB431542 (Tocris) for 6 days. Subsequently, iMNP cells were cultured in a suspension of motor neuron induction medium containing 0.5 µM RA and 0.1 µM Pur to induce pluripotent stem cell-derived motor neuron (iMN) differentiation for an additional 6 days. For mature motor neuron differentiation, iMN cells were cultured with 0.5 µM RA, 0.1 µM Pur, and 0.1 µM Compound E (Calbiochem, San Diego, CA, USA) for 10 days.

#### Immunofluorescence (IF) staining for in vitro assay

To assess BDNF expression, 5 × 10^4^ MSC and BDNF-eMSC were seeded onto coverslips in a 12-well plate. IF staining for MSC and BDNF-eMSC were performed 2 and 7 days after cell seeding, respectively. Cell seeding was performed on a 12-well plate laminin (10 mg/ml)-coated coverslip for IF staining in motor neuron cells. In iNEP, 5 × 10^4^ cells were seeded, and for iMNP, iMN, and iPSC-derived mature motor neuron (iMature MN) cells were seeded with 5 × 10^5^ cells on a laminin-coated coverslip. IF staining for iNEP, iMNP, and iMN were performed 6 days after cell seeding, whereas iMature MN was stained after 10 days. The IF staining protocol for BDNF expression and motor neuron differentiation were performed under the same conditions. All cells were fixed in 4% PFA for 30 min at RT and permeabilized using 0.1% Triton X-100 for 20 min at RT. Cell blocking was performed using PBS containing 2% BSA (PBA, Sigma-Aldrich) for 30 min. Primary antibodies were incubated with 2% PBA for 2 h at RT. After washing the cells using tris-buffered saline (TBS) with 0.05% Tween-20 (TBST), secondary antibodies conjugated with Alex Fluor-488 or 594 (Life Technologies) were incubated with 2% PBA for 1 h at RT. The stained cells were counterstained with 4′, 6-diamidino-2-phenylindole (DAPI, Roche, Basel, Switzerland), washed, and mounted using an antifade mounting reagent (Thermo Fisher Scientific). The stained cells were observed under a fluorescence LSM 900 and FV 3000 confocal microscope (Carl Zeiss, Oberkochen, Germany and Olympus Life science (EVIDNT), Tokyo, Japan ) (x 200 magnifications). The intensity of IF staining was measured in four areas at 200× magnification. The measured fluorescence intensity was analyzed using Fiji (Windos-64 Image J). Table 1 details the primary antibodies.

**Table 1:** Primary antibodies are summarized. List of Antibodies, Host, and Dilutions information

Primary Antibody	Host	Dilution	Manufacturer
Growth factors			
BDNF	Rabbit	1:50 †	GeneTex, Abcam
		1:700 *	GeneTex, Abcam
Motor neuron differentiation			
SOX1	Goat	1:50 †	R&D SYSTEMS
		1:700 *	
OLIG2	Rabbit	1:50 †	Millipore
		1:700 *	
HB9	Rabbit	1:50 †	Millipore
		1:700 *	
SMI-32	Mouse	1:50 †	BioLegend
		1:700 *	
Oligodendrocyte and Neuron			
CC-1 (Oligodendrocyte)	Mouse	1:100 †	Merck
		1:700 *	
Neu N (Neuron)	Mouse	1:100 †	Abcam
		1:700 *	
Synaptic connections and neural network			
MAP-2	Mouse	1:50 †	Santa cruz
		1:700 *	
GAP-43	Mouse	1:50 †	Santa cruz
		1:700 *	
Synapsin-1	Rabbit	1:50 †	Sigma-Aldrich
House Keeping Protein			
Beta actin	Rabbit	1:2000 *	Abcam

* Dilution of immunofluorescence staining (Dilution: )

† Dilution of western blot (Dilution: )

#### 2-dimensional (2D) and 3-dimensional (3D) co-culture and in vitro neurite outgrowth assay

We performed BDNF-eMSC and iMN co-culture to analyze BDNF expression, mature motor neuron differentiation, and neurite outgrowth in vitro. For BDNF expression and mature motor neuron differentiation, BDNF-eMSC and iMN cells were cultured at a 1:1 ratio in a 2D co-culture. Mature motor neurons were differentiated for 10 days after 2D co-culture cell seeding on a laminin-coated plate. We used a 3D co-culture platform to assess neurite outgrowth cells during mature motor neuron differentiation. We generated BDNF-eMSC and iMN 3D aggregates using microwell plates (AggreWell ^TM^ 800, STEMCELL Technologies, Seattle, WA USA) following the manufacturer’s instructions. After aggregating BDNF-eMSC and iMN in the aggrewell for 2 days, the 3D-co-culture spheroids were attached to a laminin-coated plate and differentiated into mature motor neurons for 10 days. The neurite outgrowth during mature motor neuron differentiation was confirmed and evaluated using microtubule-associated protein-2 (MAP-2) and a neurite outgrowth assay kit (Life Technologies). Neurite outgrowth was evaluated using a fluorescence plate reader. Red fluorescence was detected using emission settings of 554/567 nm for the neurite outgrowth cells. To analyze synaptic connections and neural networks in mature motor neurons, IF staining was performed using synapsin-1, Tuj-1, and MAP-2 antibodies. Fluorescence intensity was measured in four areas at 200× magnification.

#### MEA analysis

Electrophysiological analysis of mature motor neurons was performed using MEA. The 3D spheroid was made using AggreWell. Before cell seeding, the MEM plate was coated with 0.1% polyethyleneimine solution for 1 h at RT, followed by rinsing with sterile deionized water thrice and dried overnight in a biosafety cabinet at RT. The 3D spheroids per AggreWell treated with laminin (10 µg/mL) were seeded onto the MEA plate. After 1 h, the 3D spheroids were incubated with a mature motor neuron induction medium. The MEA plate was placed in a cell culture incubator with a 5% CO_2_ humidified atmosphere at 37 ℃. Electroactivity of neurons was monitored after 10 days of culture and the number of spikes was recorded.

#### Protein extraction in cell lysates and WB analysis

Protein was extracted from BDNF-eMSC, MSC, and iMNP cells using RIPA buffer (Thermo Fisher Scientific). The cell lysate was obtained after 10 days of 2D co-culture to analyze the expressions of BDNF and mature motor neuron marker, as well as mature motor neuron differentiation through 2D BNDF-eMSC and iMN culture. The cell lysate was incubated with RIPA buffer for 30 min at 4℃, followed by centrifugation at 16,000 rpm for 20 min. The amount of extracted protein was quantified using a bicinchoninic acid (BCA) protein assay. To confirm the expression of BDNF, motor neuron differentiation markers and MAP-2 in the quantified protein supernatant were separated using sodium dodecyl sulfate-polyacrylamide gel electrophoresis and transferred onto a nitrocellulose blotting membrane. The membrane was blocked with 3% BSA for 1 h at RT and incubated with primary antibodies overnight at 4℃. The following day, the membrane was incubated with secondary antibodies at RT for 1 h. Subsequently, protein expression was confirmed using an enhanced chemiluminescence solution. Protein expression was detected using LAS 4000 (BioRad, Herecules, CA, USA), and band intensity was quantified using multi-gauge V 3.0 software (Fujifilm, Tokyo, Japan). Full-length Western blot images are presented in Additional file 4: Fig. 4.

### In vivo assay

#### Animal care and contusive chronic SCI model

The Animal Studies Committee of the School of Medicine, the Catholic University of Korea, approved this study (IACUC approval Number CUMC-2020-0364-04). All animal care, operation, and cell transplantation procedures were conducted in accordance with the Laboratory Animal Welfare Act and the Guideline and Policies for Rodent Survival Surgery. The Animal Research: Reporting of In Vivo Experiments (ARRIVE) guidelines were followed. Contusive chronic SCI models were generated and prepared based on a previously reported surgical procedure [16, 30]. Briefly, a contusive chronic SCI model was generated using 7-week-old adult male Sprague-Dawley rats (weighing between 270 and 320 g). The rats were anesthetized with isoflurane via inhalation and Rompun (2 mg/kg) via intraperitoneal injection. After anesthesia, the rats were shaved and sterilized with antiseptic betadine. The paravertebral muscles from thoracic 8 and 10 (T8–10) were exposed, and a laminectomy was performed at T9. The contusive SCI model was induced using the Multicenter Animal Spinal Cord Injury Study impactor (a 10 g rod was dropped from a height of 2.5 cm) during the laminectomy at T9. Pre- and post-operatively, the rats were administered 5 mg of ketoprofen, gentamicin, and warm saline solution for 3–5 days. The bladders of all rats with SCI were manually emptied for 1 week. Behavioral recovery was observed for 6 weeks after SCI, and rats that spontaneously recovered were excluded before combination cell transplantation.

#### Group allocation and combination cell transplantation

The concepts of combination cell transplantation have been performed in the induced contusive chronic SCI model. We performed and recorded the behavioral assessment before cell transplantation. The rats were randomized into the following groups at 6 weeks post-SCI for cell transplantation: (1) chronic SCI + phosphate-buffered saline (PBS) group (*n* = 8), (2) chronic SCI + BDNF-eMSC group (*n* = 6), (3) chronic SCI + iMNP group (*n* = 7), and (4) chronic SCI + BDNF-eMSC + iMNP group (*n* = 8). Before cell transplantation, BDNF-eMSCs were labeled with PKH26 (red fluorescence), whereas iMNPs were labeled with PKH67 (green fluorescence). At 6 weeks post-injury, the lesion site (T9) was re-exposed, and 1 × 10^6^ cells of BDNF-eMSC and iMNP cells in 10 µL PBS were transplanted using a Hamilton needle in the BDNF-eMSC and iMNP groups, respectively. The BDNF-eMSC + iMNP group was transplanted with both types of cells (1:1) in 10 µL PBS at the lesion site. The PBS group was transplanted with 10 µL PBS at the lesion site. All groups were transplanted at the rostral (5 µL) and caudal (5 µL) of the lesion site. We intramuscularly administered 10 mg/kg of cyclosporin A (Cipol Inj, Chongkundang Pharmaceutical) daily after cell transplantation.

#### Cell labeling for tracking transplanted cells

The transplanted cells were tracked, and their engraftment and differentiation in the injured SC were confirmed using PKH26 (red fluorescence) (Sigma-Aldrich, St Louis, MO, USA) and PKH67 (green fluorescence) (Sigma-Aldrich). PKH26 and PKH67 are fluorescent cell membrane-intercalating dyes. Before cell transplantation, BDNF-eMSC was labeled with PKH26, whereas iMNP was labeled with PKH67. Briefly, the PKH26 and PKH67 cell tracking procedure was the same. A cell pellet containing 1 × 10^6^ cells was incubated with Diluent C and cell tracking dye solutions for 5 min at room temperature (RT). After labeling, the activity of the cell tracking dyes was stopped using 1% bovine serum albumin (BSA). After the final wash, the cell pellet was centrifuged and suspended in PBS for cell transplantation.

#### Behavioral recovery assessment

We assessed behavioral recovery using the Basso, Beattie, and Bresnahan (BBB) locomotor rating scale after SCI. Three researchers monitored the BBB locomotor scale and recorded the scores every week for 12 weeks. Rats that exhibited natural, spontaneous improvement of hindlimb function within 24 h post-injury were excluded. Furthermore, rats that spontaneously recovered before cell transplantation were excluded. Based on the BBB locomotor scales, rats were divided into two grades: grade 1 (0–5) and grade 2 (6–11). Improvements in behavioral recovery were compared using the incidence rate. The incidence rate (%) was calculated as follows: (BBB grade score of total rats/total rats) x 100.

#### Preparation of injured SC tissue

We euthanized the specimens using CO_2_ gas (30–70% chamber volume/min) before obtaining the injured SC, following the American Veterinary Medical Association Guidelines for the Euthanasia of Animals (2020 Edition). The injured SC samples (approximately 1 cm segment) were obtained at 12 weeks post-injury. For immunofluorescence (IF) staining assessments, we first confirmed cardiac arrest, and then the injured SC was obtained after trans-cardiac perfusion with PBS and 4% paraformaldehyde (PFA). The obtained injured SC was fixed overnight in 4% PFA, followed by overnight incubation in 15% and 30% sucrose at 4℃. Injured SC was embedded in optical cutting temperature (Tissue-Tek; Sakura Finetek USA, Torrance, CA, USA) and snap-frozen using liquid nitrogen. For Western blot (WB) analysis, injured SC (approximately 1 cm segment) was obtained after euthanasia without a prior cardiac perfusion procedure. The obtained injured SC was immersed in liquid nitrogen and stored in a deep freezer at -80℃.

#### IF staining for injured SC

IF staining was performed on frozen sections of the embedded injured segments (approximately 1 cm) to assess transplanted cell engraftment, differentiations, axonal regeneration, and BDNF expression. Frozen SC sections (4 μm thick) were obtained and mounted on saline-coated slides. The SC sections were fixed using cold acetone for 10 min at RT, followed by washing with TBST. The slide sections were permeabilized using 0.1% Triton X-100 for 20 min at RT. Subsequently, the SC sections were blocked with normal goat or horse serum containing 0.1% Triton X-100 for 1 h at RT. Primary antibodies were incubated with 0.1% Triton X-100 and 1% normal goat or horse serum overnight at 4℃. After washing, Cy5 (Life Technologies, Carlsbad, CA, USA) Fluor-conjugated secondary antibodies were incubated and diluted in TBST at RT for 1 h. Slides were washed, stained with DAPI, and finally washed and mounted using an antifade mounting reagent. We confirmed and observed the IF staining under a confocal microscope, LSM 700 and 900 and FV 3000 (Carl Zeiss, Oberkochen, Germany) and Olympus Life science (EVIDNT). The number of engrafted cells in the lesion site was counted in six areas at 400× magnification for PKH26 (BDNF-eMSC) and PKH67 (iMNP). Transplanted cells were counted using the cell counter plug-in of Fiji (Windows-64 Image J) program. The intensity of engrafted cells was measured in six areas at 400× magnification. The intensity of IF staining was measured in four areas at x200 magnification. The measured fluorescence intensity was analyzed using Fiji (Windows-64 image J) program. Table 1 details the primary antibodies.

#### Protein extraction in injured spinal cord and WB analysis

The injured SC segments (approximately 1 cm length) were extracted using tissue protein extraction reagent (Thermo Fisher Scientific) with one protease inhibitor cocktail tablet (Roche, Basel, Switzerland) and 1 mM phenyl methyl sulfonyl fluoride for 1 h at 4℃. Additionally, the tissue-extracted protein amount was quantified using a BCA quantitative analysis. Protein quantification was separated using sodium dodecyl sulfate-polyacrylamide gel electrophoresis and transferred onto a nitrocellulose blotting membrane. The transferred membranes were blocked using 3% BSA in TBST for 1 h at RT and incubated with primary antibodies overnight at 4℃. Table 1 detail the primary antibodies. Membranes were incubated with secondary peroxidase-conjugated antibodies for 1 h at RT. Protein expression was confirmed using an enhanced chemiluminescence solution. Protein expression was detected via exposure to LAS 4000 (BioRad, Hercules, CA, USA), and band intensity was quantified using multi-gauge V 3.0 software (Fujifilm, Tokyo, Japan). Full-length western blot images are presented in Additional file 4: Fig. 4.

### Statistical analysis

All results were statistically analyzed using IBM SPSS statistics for Windows, version XX (IBM Corp., Armonk, N.Y., USA). All data were expressed as means ± standard deviations. For BDNF expression analysis in hMSC and BDNF-eMSC in vitro assay, the paired t-test (#) and Kruskal–Wallis test followed by Mann–Whitney U test (†) were used to compare the results between the two groups. Statistical significance was set at a *p* < 0.05. The statistical relevancies were expressed using a one-way analysis of variance and Fisher’s least significant difference (*) and Kruskal–Wallis tests followed by Mann–Whitney U test for intergroup comparison to compare the results among the three or four groups. Statistical significance was set at *p*<0.05 (†*# *p* < 0.05, ††**## *p* < 0.01, †††***### *p* < 0.001, n.s = not significant).

## Results

### BDNF over-expressing engineered mesenchymal stem cell (BDNF-eMSC) generation

**Fig. 1:**
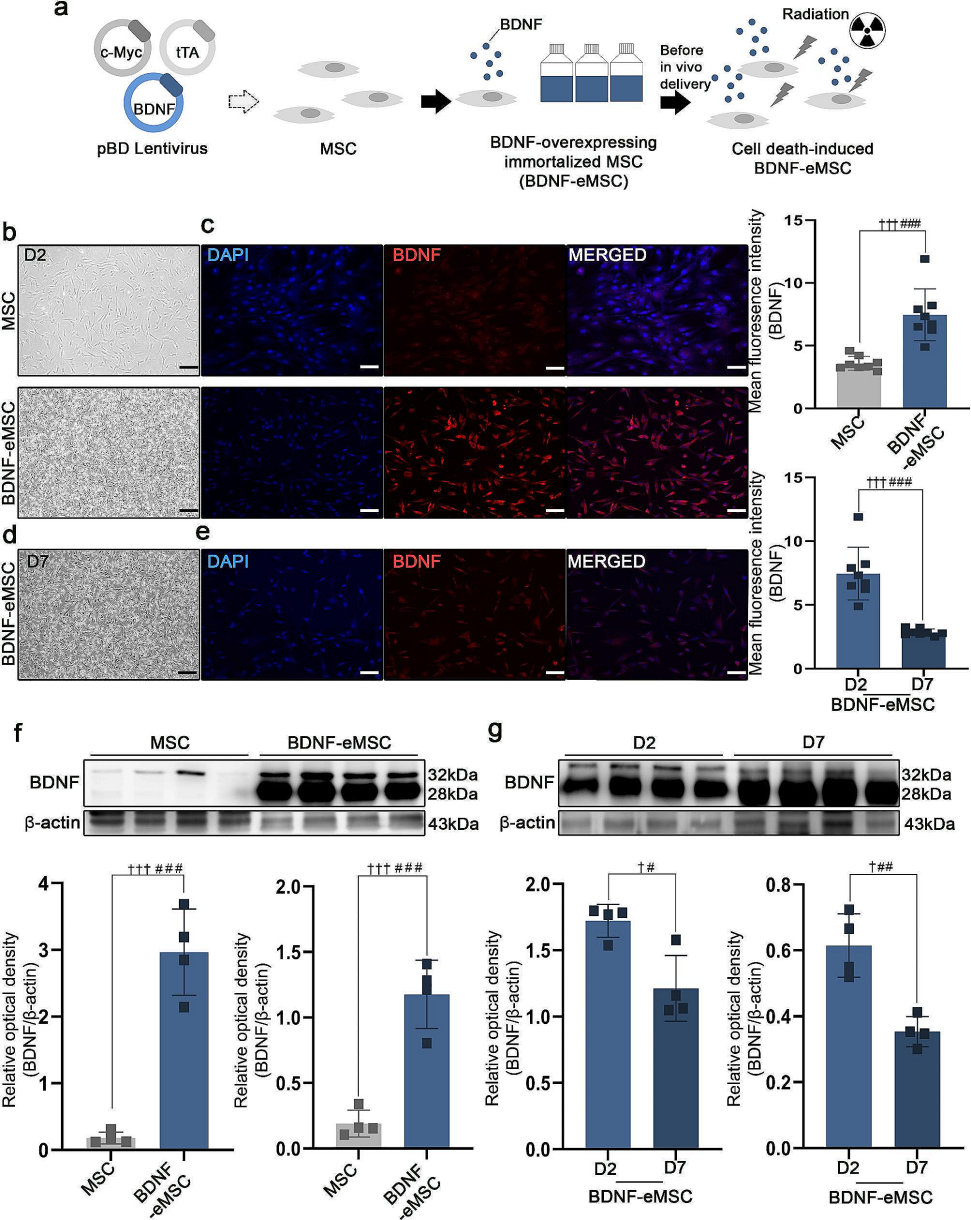
Generation of BDNF-eMSCin vitro. (**a**) The scheme of BDNF-eMSC. (**b**) Representative light microscope images of MSC and BDNF-eMSC cell morphologies on day 2. (**c**) Representative fluorescence images showing BDNF expression in MSC and BDNF-eMSC on day 2. Quantification of the fluorescence intensity of BDNF in MSC and BDNF-eMSC (*n* = 8). (**d**) Representative light microscope image depicting BDNF-eMSC morphology on day 7. (**e**) Representative fluorescence image showing BDNF expression on BDNF-eMSC on day 7. Quantification of the fluorescence intensity of BDNF in BDNF-eMSC on day 2 and 7 (*n* = 8). (**f**) Western blotting (WB) results showing BDNF expression in MSC and BDNF-eMSC after 2 days of cell lysates. (**g**) WB results demonstrating BDNF expression in BDNF-eMSC after 2 and 7 days of cell lysates. Full-length western blot images are presented in Additional file 4: Fig. 4. The data are presented as mean ± SEM. Statistical significance was estimated using paired t-test (#) and Kruskal–Wallis analysis followed by Mann–Whitney (†) analysis for intergroup comparison. #, † *P* < 0.05, ## *P* < 0.01. ### *P* < 0.001. (MSC: 2 days *n* = 4, BDNF-eMSC: 2 days *n* = 4, BDNF-eMSC: 7 days *n* = 4). Scale bars = 50 μm. BDNF-eMSC, BDNF over-expressing engineered mesenchymal stem cells; IF, Immunofluorescence staining; WB, Western blot

In previous studies reported that the BDNF-eMSC demonstrated highly proliferative and secreted BDNF expression than naïve MSCs. BDNF-eMSC was also increased the BDNF expression than naïve MSCs. The BDNF-eMSC was generated as previously described [17–19]. Before cell transplantation, we confirmed and performed BDNF expression in BDNF-eMSC. The BDNF-eMSC was established using a lentiviral vectors encoding the c-Myc, tTA and BDNF genes and then irradiated with 200 Gy radiation using an X-ray irradiation device (Red Source Technologies, Buford, GA, USA) (Fig. 1a). On day 2 after cell thawing, the BDNF-eMSC displayed a homogenous spindle-like cell morphology representative of MSCs (Fig. 1b). MSC and BDNF-eMSC exhibited positive expression of BDNF on day 2. The fluorescence intensity of BDNF was statistically significantly higher in BDNF-eMSC than in MSC (Fig. 1c). BDNF-eMSC maintained their spindle-like cell morphology till day 7 (Fig. 1d). IF staining revealed BDNF expression in BDNF-eMSC on day 7. However, the fluorescence intensity of BDNF in BDNF-eMSC significantly decreased on day 7 compared to that on day 2 (Fig. 1e). WB analysis confirmed higher BDNF expression in BDNF-eMSC than in MSCs (Fig. 1f). Furthermore, we observed that BDNF expression in BDNF-eMSC decreased on day 7 than on day 2 (Fig. 1g). Full-length western blot images are presented in Additional file 4: Fig. 4.

### Human iPSC derived motor neuron progenitor and motor neuron differentiation

**Fig. 2:**
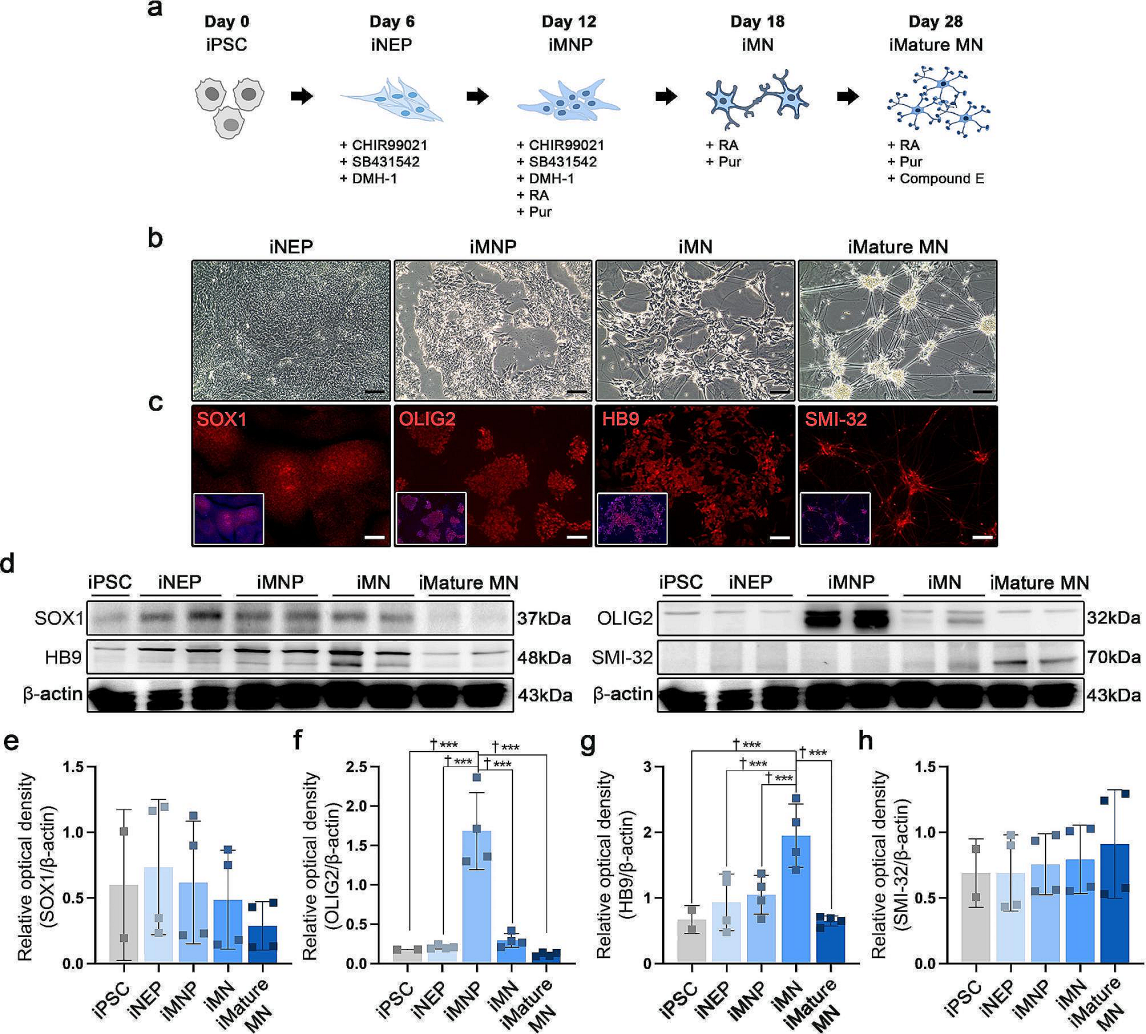
Generation of induced pluripotent stem cell (iPSC)-derived motor neurons using small moleculesin vitro. (**a**) The scheme of motor neuron progenitor cells (MNP), motor neuron (MN), and mature MN differentiation from human iPSCs using a small molecule cocktail. (**b**) Representative time course light microscopy images displaying induced pluripotent stem cell-derived neuron epithelial progenitor cell (iNEP), induced pluripotent stem cell-derived motor neuron progenitor cells (iMNP), induced pluripotent stem cell-derived motor neuron cells (iMN), and induced pluripotent stem cell-derived mature motor neuron cells (iMature MN) differentiation. (**c**) Fluorescence time course images of iNEP, iMNP, iMN, and iMature MN using stage differentiation markers. (**d-h**) WB results of motor neuron stage differentiation markers of stage cell differentiation in cell lysates. (iNEP: *n* = 4, iMNP; *n* = 4, iMN: *n* = 4, and iMature MN: *n* = 4). Full-length western blot images are presented in Additional file 1: Fig. 1. The data are presented as mean ± SEM. Statistical significance was estimated using the Kruskal–Wallis test with *post hoc* analysis and the Mann–Whitney (†) test with the least significant difference *post hoc* analysis (*); *, † *P* < 0.05, **†† *p* < 0.01. Scale bars = 50 μm. iPSCs, induced pluripotent stem cell; iNEPs, induced pluripotent stem cell-derived neuron epithelial progenitor cells; iMNPs, induced pluripotent stem cell-derived motor neuron progenitor cells; iMNs, induced pluripotent stem cell-derived motor neuron cells; iMature MNs, induced pluripotent stem cell-derived mature motor neuron cells; IF, Immunofluorescence staining; WB, Western blot. SOX1 = iNEP, OLIG2 = iMNP, HB9 = iMN, SMI-32 = iMature MN.

The motor neuron progenitor and mature motor neurons were differentiated using a small molecule cocktail as previously described [28, 29] (Fig. 2a). Our results revealed that iMNP and iMature MN were successfully differentiated and reproduced from human iPSC, as confirmed via light microscopy (Fig. 2b). We evaluated the stages of motor neuron differentiation using the specific marker expression of each cell differentiation phase. On day 6, IF staining and WB analysis revealed Sox 1 expression in the iNEP phase (Fig. 2c and d-e). The fluorescence intensity of SOX1 was significantly higher in iNEP (Additional file 1: Fig. 1). OLIG2 expression was more strongly expressed in the iMNP phase than in other motor neuron cell differentiation stages (Fig. 2d and f). The fluorescence intensity of OLIG2 was also significantly higher in iMNP (Additional file 1: Fig. 1). IF staining revealed HB9-positive cells in the iMN phase (Fig. 2c), and the fluorescence intensity of HB9 was significantly higher in iMN (Additional file 1: Fig. 1). The protein expression increased on day 18, as confirmed by WB analysis (Fig. 2d and g). In the iMature MN phase, we evaluated SMI-32 expression using IF staining and WB analysis. The fluorescence intensity of SMI-32 was statistically significantly higher in mature iMNs than in iNEP, iMNP, and iMN (Additional file 1: Fig. 1). On day 28, iMature MN showed increased SMI-32 positivity and protein expression (Fig. 2c and d-h). Full-length western blot images are presented in Additional file 4: Fig. 4.

### Combination cell transplantation enhances behavioral improvement in a contusive chronic SCI model

**Fig. 3:**
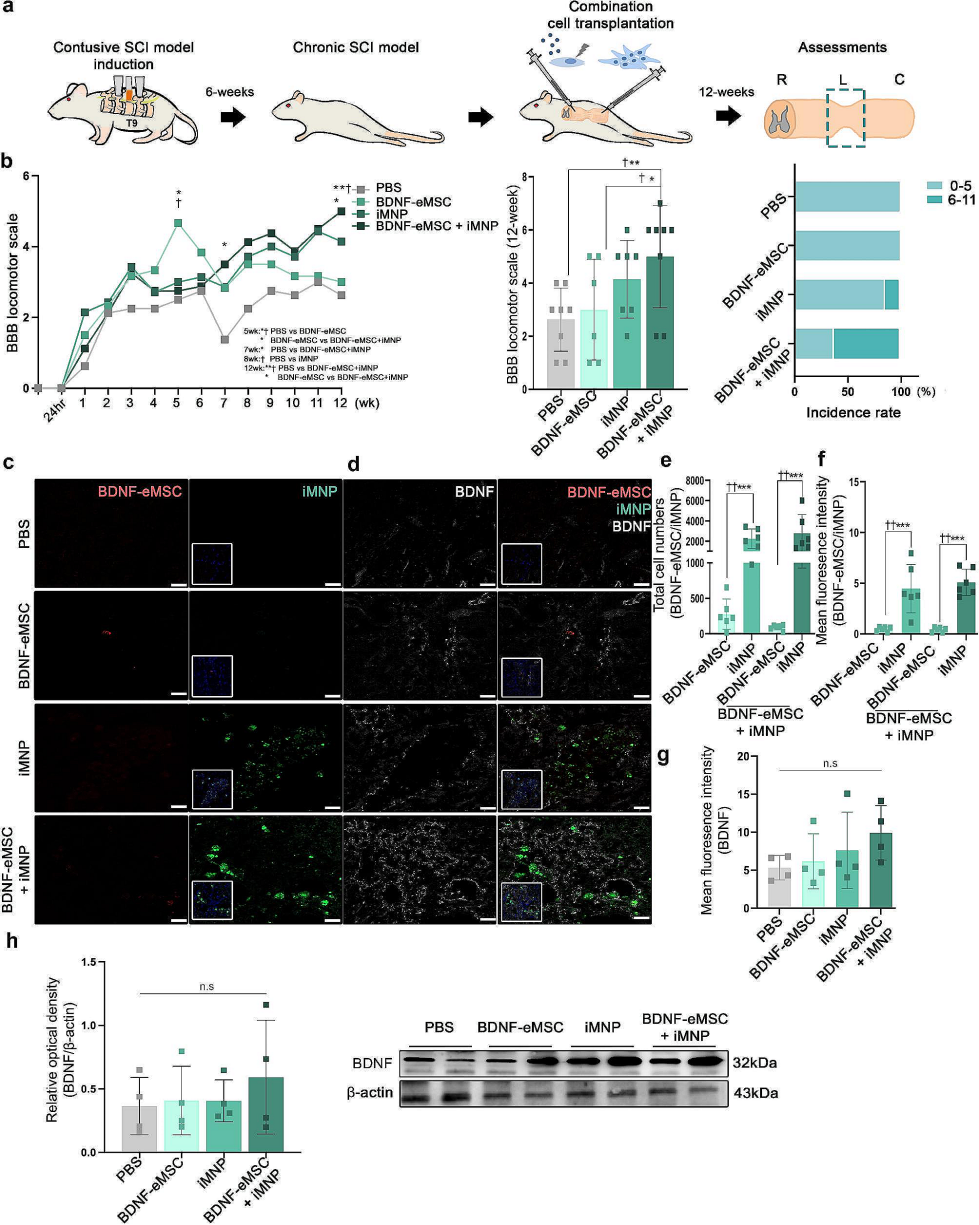
Combined transplantation of BDNF-eMSC and iMNP in a contusive chronic SCI Model. (**a**) Experimental schemes illustrating contusive chronic SCI rat model generation, combined cell transplantation, and clinical behavior and histology assessments. (**b**) The BBB scales and incidence rates 12 weeks after SCI (PBS: *n* = 8, BDNF-eMSC: *n* = 6, iMNP: *n* = 7, BDNF-eMSC + iMNP *n* = 8). (**c**) IF images showing merged 4´,6-diamidino-2-phenylindole and transplanted cells (red; BDNF-eMSC, green; iMNP) at the lesion site at 12 weeks. (**d**) Multi-fluorescent confocal images showing the expression of BDNF and transplanted cells in the white matter. (**e**) The number of engrafted cells at the lesion site (*n* = 6). (**f**) Quantification of the fluorescence intensity of engrafted cells at the lesion site (*n* = 6). (**g**) Quantification of the fluorescence intensity of BDNF at the lesion site (*n* = 4). (**h**) WB results of BDNF expression at the lesion site segments (approximately 1 cm). Full-length western blot images are presented in Additional file 4: Fig. 4. The data are presented as mean ± SEM. Statistical significance was estimated using the Kruskal–Wallis test with *post hoc* analysis and Mann–Whitney U (†) test with least significant difference *post hoc* analysis (*); *, † *P* < 0.05, **†† *p* < 0.01. (PBS: *n* = 4, BDNF-eMSC: *n* = 4, iMNP: *n* = 4, BDNF-eMSC + iMNP: *n* = 4). Scale bars = 50 μm. BDNF-eMSC, BDNF over-expressing engineered mesenchymal stem cells; BBB, Basso–Beattie–Bresnahan; iMNPs, induced pluripotent stem cell-derived motor neuron progenitor cells; IF, Immunofluorescence staining; WB; Western blot

Before cell transplantation, BDNF-eMSC was labeled with PKH 26 (red), whereas iMNP cells were labeled with PKH67 (green). First, we generated a contusive chronic SCI model and transplanted BDNF-eMSC and iMNP cells into the injured SC via the intralesional route at 6 weeks post-injury. At 12 weeks post-injury, we assessed the engraftment of transplanted cells and BDNF expression in the injured SC (Fig. 3a). We used the BBB locomotor scales to evaluate the clinical recovery of behavior for 12 weeks post-injury. At 12 weeks post-injury, the BDNF-eMSC + iMNP group exhibited a significantly improved functional recovery than the PBS and BDNF-eMSC groups. The incidence ratio of BBB score 6–11 was 62.5% in the BDNF-eMSC + iMNP group and 14.28% in the iMNP group at 12 weeks post-injury (Fig. 3b and Supplementary Movie 1, 2, 3 and 4). At 12 weeks post-injury, transplanted BDNF-eMSC (PKH26, red) were not observed in the white and gray matter of the lesion site. In contrast, transplanted iMNP (PKH67, green) and BDNF-eMSC + iMNP (PKH67, green) cells were observed and persistent in the white and gray matter of the lesion (Fig. 3c and e-f). However, fewer BDNF-eMSC + iMNP cells were observed in the transplanted BDNF-eMSC at the lesion site (Fig. 3c and e-f). At 12 weeks after injury, iMNP cells rather than BDNF-eMSCs remained at the lesion site (Fig. 3c and e-f). However, it was confirmed that BDNF-eMSCs engrafted and remained at the lesion site one week after transplantation (Additional file 2: Fig. 2). BDNF expression was assessed using IF staining to confirm BDNF expression at the lesion site. All cell transplantation groups showed BDNF expression compared with the PBS group at 12 weeks post-injury. The IF staining showed the BDNF-eMSC + iMNP group had a higher fluorescence intensity than the PBS, BDNF-eMSC, and iMNP groups. However, significant differences were not observed between the groups (Fig. 3d and g). In the WB analysis, the BDNF-eMSC + iMNP group had a higher BDNF expression than the PBS, BDNF-eMSC, and iMNP groups at the lesion segment (approximately 1 cm). However, significant differences were not observed between all groups (Fig. 3h). Full-length western blot images are presented in Additional file 4: Fig. 4.

### BDNF-eMSC + iMNP combination cell transplantation promotes motor neuron maturation and growth density of neuronal processes at the lesion site

**Fig. 4:**
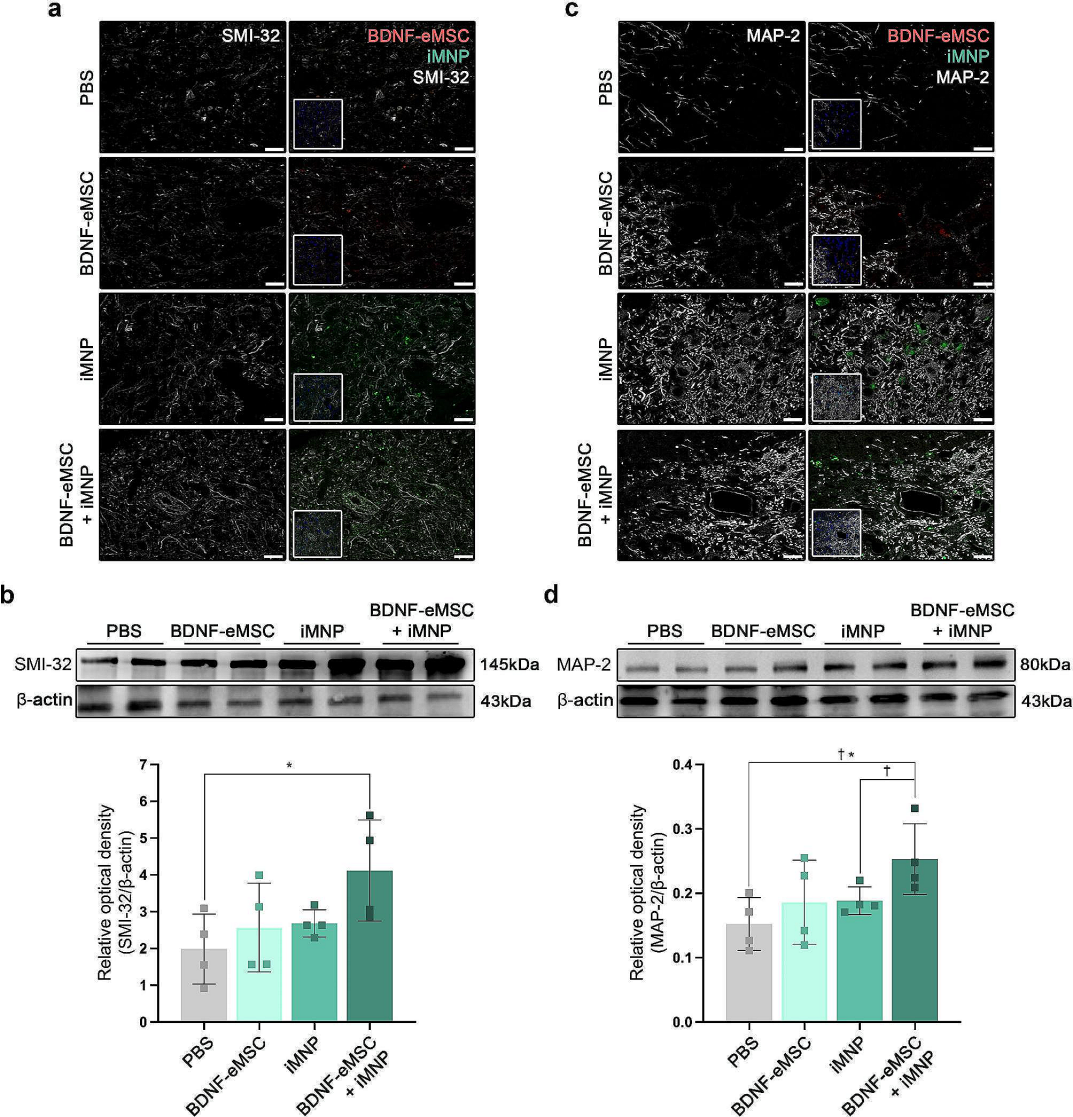
Enhancements of mature MN differentiation and growth density of neuronal processes by BDNF-eMSC and iMNPin vivo. (**a**) IF images showing the merged SMI-32 and transplanted cells at the lesion site, with SMI-32 differentiation of the transplanted iMNP cells being predominant in the lesion site at 12 weeks post-injury. (**b**) WB results of SMI-32 expression at the lesion site segment (approximately 1 cm) (PBS: *n* = 4, BDNF-eMSC: *n* = 4, iMNP: *n* = 4, BDNF-eMSC + iMNP: *n* = 4). Full-length WB images are presented in Additional file 1: Fig. 1. (**c**) Confocal images showing MAP-2 and transplanted cell expression around the lesion site. (**d**) WB results of MAP-2 expression at the lesion site segment (approximately 1 cm) (PBS: *n* = 4, BDNF-eMSC: *n* = 4, iMNP: *n* = 4, BDNF-eMSC + iMNP *n* = 4). Full-length western blot images are presented in Additional file 4: Fig. 4. The data are presented as mean ± SEM. Statistical significance was estimated using the Kruskal–Wallis test with *post hoc* analysis and the Mann–Whitney (†) test with the least significant difference *post hoc* analysis (*); *, † *P* < 0.05. Scale bars = 50 μm. BDNF-eMSC, BDNF over-expressing engineered mesenchymal stem cells; iMNP, induced pluripotent stem cell-derived motor neuron progenitor cells; IF, Immunofluorescence staining; WB, Western blot

IF staining and WB analysis confirmed motor neuron differentiation and axonal regeneration of the transplanted cells at the lesion site. Mature motor neuron differentiation was evaluated using the SMI-32 marker. At 12 weeks post-injury, SMI-32 expression was observed around the gray matter of the lesion site. The expression of SMI-32 and transplanted iMNP cells were higher in the iMNP and BDNF-eMSC + iMNP groups than that in the PBS and BDNF-eMSC groups (Fig. 4a). SMI-32 protein expression in the injured segments of the BDNF-eMSC + iMNP group significantly increased than that of the PBS group. However, significant differences were not observed between cell transplantation groups (Fig. 4b). MAP-2 marker expression was analyzed in the axial section of the injured SC to confirm axonal regeneration of the transplanted cells at the lesion site 12 weeks post-injury. MAP-2 expression was observed around the dorsal horn and central canal of the lesion site. MAP-2 expression in the transplanted iMNP and BDNF-eMSC + iMNP cells were predominant in the injured SC (Fig. 4c). MAP-2 revealed a significantly higher expression in the BDNF-eMSC + iMNP group compared with the PBS and iMNP groups (Fig. 4d). The growth density of the neuronal process at the lesion site was analyzed using the GAP-43 marker at 12 weeks post-injury. GAP-43 expression was observed around the dorsal horn and central canal of the lesion site (Additional file 3: Fig. 3a). The IF staining showed the fluorescence intensity of the cell transplantation groups was significantly higher than that of PBS (Additional file 3: Fig. 3b). The expression of GAP-43 was significantly higher in the iMNP and BDNF-eMSC + iMNP groups than in the PBS group (Additional file 3: Fig. 3c). Full-length western blot images are presented in Additional file 4: Fig. 4.

### BDNF-eMSC + iMNP combination cell transplantation increases oligodendrocyte and neuronal cell differentiation at the lesion site

**Fig. 5:**
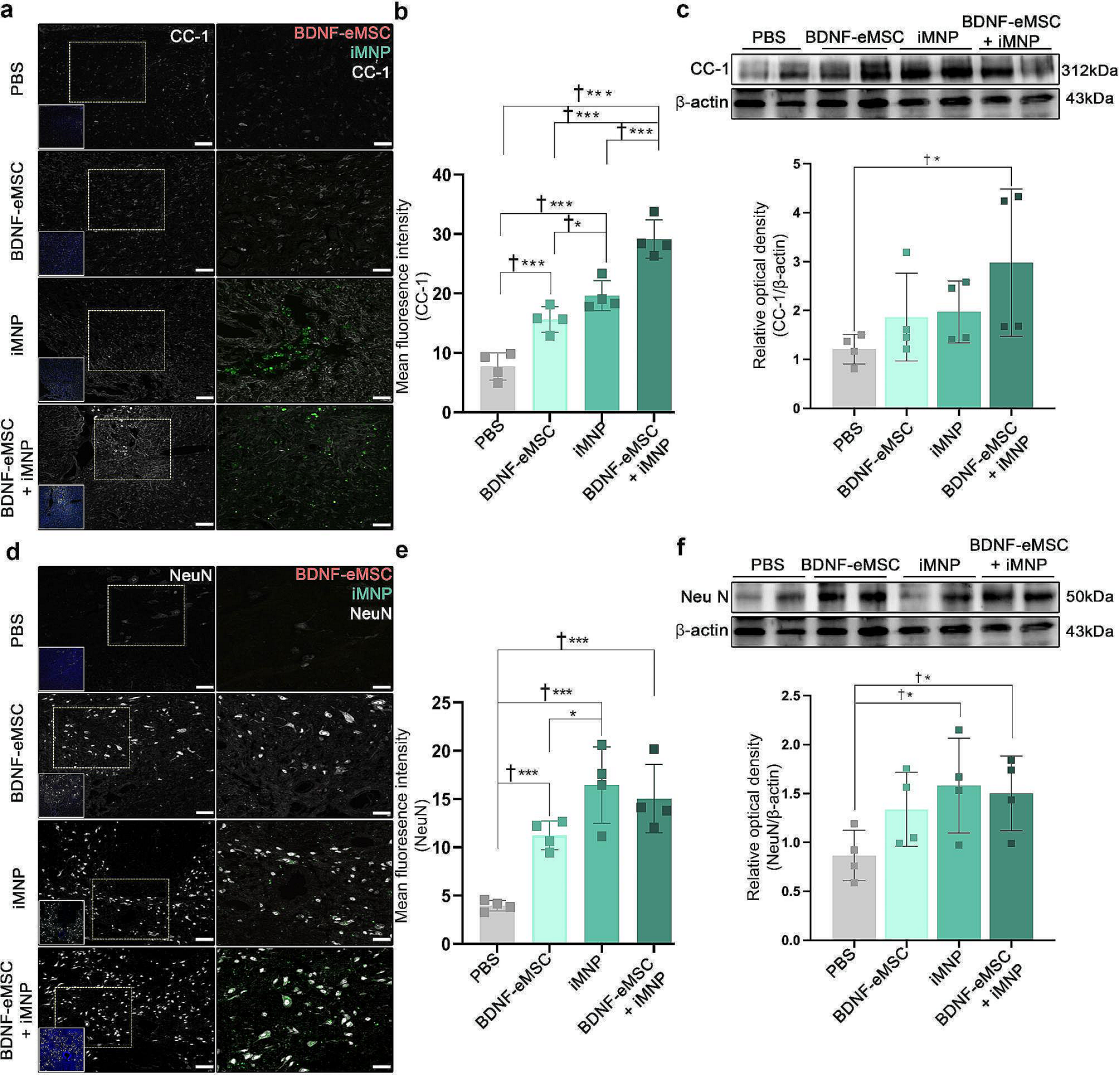
Increased oligodendrocyte and neuronal cells by BDNF-eMSC and iMNP in the lesion site. (**a**) IF image showing oligodendrocyte differentiation by transplanted BDNF-eMSC and iMNP around the injured site. (**b**) Quantification of the fluorescence intensity of CC-1 at the lesion site (*n* = 4) (**c**) WB results of CC-1 expression in the injured segment (approximately 1 cm) (PBS: *n* = 4, BDNF-eMSC: *n* = 4, iMNP: *n* = 4, BDNF-eMSC + iMNP: *n* = 4). Full-length WB images are presented in Additional file 4: Fig. 4. (**d**) IF analysis of NeuN expression, merged with transplanted cells, was observed in the gray matter. (**e**) Quantification of the fluorescence intensity of NeuN at the lesion site (*n* = 4). (**f**) Expression of neuronal cell marker NeuN, confirmed in the injured segment (approximately 1 cm) via WB. Full-length western blot images are presented in Additional file 4: Fig. 4. The data are presented as mean ± SEM. Statistical significance was estimated using the Kruskal–Wallis test with *post hoc* analysis and the Mann–Whitney (†) test with the least significant difference *post hoc* analysis (*); *, † *P* < 0.05. Scale bars = 50 μm. BDNF-eMSC, BDNF over-expressing engineered mesenchymal stem cells; iMNP, induced pluripotent stem cell-derived motor neuron progenitor cell; IF; Immunofluorescence staining, WB; Western blot

IF staining and WB analysis were performed to confirm the expression of oligodendrocyte and neuronal cell differentiation in the engrafted cells. We assessed oligodendrocyte and neuronal cell differentiation using the CC-1 and NeuN markers at the lesion site. CC-1 is a representative oligodendrocyte phenotype marker. At 12 weeks post-injury, CC-1 expression was observed around the white matter. Oligodendrocyte-positive cells of the transplanted iMNP and BDNF-eMSC + iMNP cells were predominant in the injured SC and were qualitatively abundant around the engrafted iMNP cells (Fig. 5a). The IF staining confirmed that the fluorescence intensity of CC-1 in the cell transplantation groups was significantly higher than that in the PBS group (Fig. 5b). The BDNF-eMSC + iMNP group had significantly higher CC-1 expression than the PBS group (Fig. 5c). NeuN is a neuronal cell phenotype marker. NeuN expression was mainly observed in the dorsal horn of the gray matter and central canal. NeuN and iMNP were highly expressed in the iMNP and BDNF-eMSC + iMNP groups than in the PBS and BDNF-eMSC groups in the dorsal horn of the gray matter and central canal at 12 weeks post-injury (Fig. 5d). The IF staining confirmed that the fluorescence intensity of NeuN in the cell transplantation groups was significantly higher than that in the PBS group (Fig. 5e). The iMNP and BDNF-eMSC + iMNP groups had significantly higher NeuN expression than the PBS group (Fig. 5f). Full-length western blot images are presented in Additional file 4: Fig. 4.

### BDNF-eMSC and iMN play a critical role in promoting motor neuron maturation and growth density of neuronal processes in vitro

**Fig. 6:**
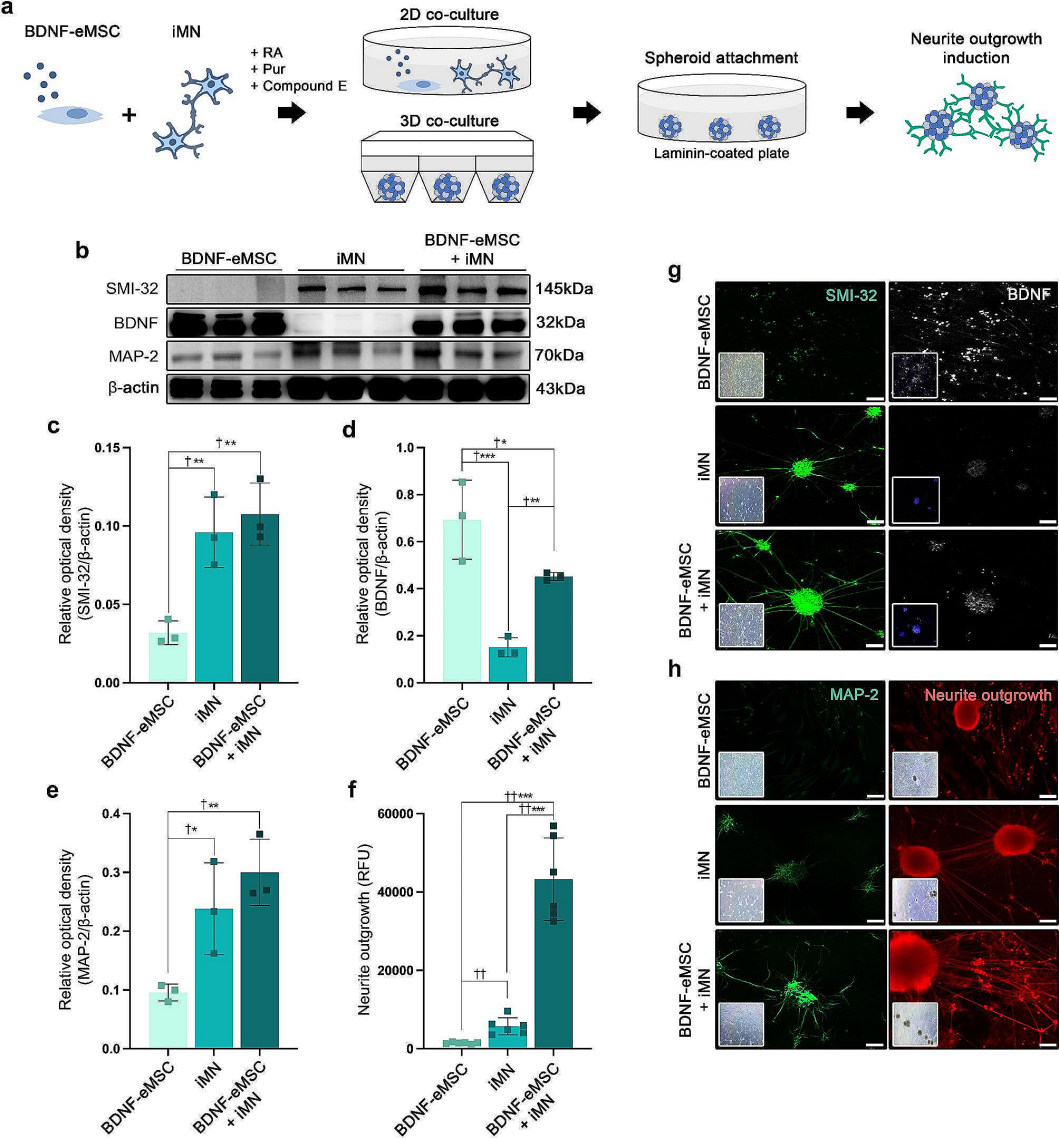
Increased MN maturation and axonal regeneration induction by BDNF-eMSC and iMN cell co-culture. (**a**) Schematic of BDNF-eMSC and iMN 2D and 3D co-culture motor neuron differentiation and maturation and axonal regeneration assessments in vitro assay. (**b**) Representative WB images of SMI-32, BDNF, MAP-2 in 2D co-culture on day 10. (**c**) Protein expression of SMI-32 in BDNF-eMSC and iMN 2D co-culture cell lysates on day 10. (**d**) Protein expression of BDNF in BDNF-eMSC and iMN 2D co-culture cell lysates on day 10. (**e**) Protein expression of MAP-2 in BDNF-eMSC and iMN 2D co-culture cell lysates on day 10 (BDNF-eMSC: *n* = 3, iMN: *n* = 3, BDNF-eMSC + iMN: *n* = 3). (**f**) Quantification of neurite outgrowths on day 10 in 3D co-cultured spheroid (BDNF-eMSC: *n* = 6, iMN: *n* = 6, BDNF-eMSC + iMN: *n* = 6). (**g**) Representative IF images of SMI-32 in BDNF-eMSC and iMN 2D co-culture on day 10. Representative IF images of BDNF in BDNF-eMSC and iMN 2D co-culture on day 10. (**h**) Representative IF images of MAP-2 in BDNF-eMSC and iMN 2D co-culture on day 10. Representative fluorescence images of neurite outgrowth on day 10 in 3D co-cultured spheroid. Full-length western blot images are presented in Additional file 4: Fig. 4. The data are presented as mean ± SEM. Statistical significance was estimated using the Kruskal–Wallis test with *post hoc* analysis and the Mann–Whitney (†) test with the least significant difference *post hoc* analysis (*); *, † *P* < 0.05, **†† *p* < 0.01, *** *p* < 0.001. Scale bars = 50 μm. BDNF-eMSC, BDNF over-expressing engineered mesenchymal stem cells; iMN, induced pluripotent stem cell-derived motor neuron; IF, Immunofluorescence staining; WB, Western blot

In vivo results demonstrated that combination cell transplantation using BDNF-eMSC and iMNP promoted motor neuron maturation and axonal regeneration at the lesion site. 2D and 3D co-culture of BDNF-eMSC and iMN were performed to confirm the effect of motor neuron differentiation and axonal regeneration in vitro based on previous in vivo results. We analyzed the motor neuron maturation and axonal regeneration by co-culturing BDNF-eMSC and iMN in 2D and 3D spheroid platforms during mature motor neuron differentiation (Fig. 6a). In the IF staining, SMI-32 expression was qualitatively higher in the iMN and BDNF-eMSC + iMN groups than in the BDNF-eMSC group (Fig. 6g). In the WB analysis, SMI-32 expression was also significantly higher in the iMN and BDNF-eMSC + iMN groups than in the BDNF-eMSC group (Fig. 6b and c). BDNF expression was qualitatively higher in the BDNF-eMSC and BDNF-eMSC + iMN groups than in the iMN group (Fig. 6g). In the WB analysis, BDNF expression was significantly higher in the BDNF-eMSC group than in the iMN and BDNF-eMSC + iMN groups. Additionally, BDNF expression was significantly higher in the BDNF-eMSC + iMN group than in the iMN groups on day 10 (Fig. 6d). The MAP-2 marker was analyzed in a 2D co-culture platform to evaluate the expression of axonal regeneration. In the IF staining and WB analysis, MAP-2 expression was significantly higher in the iMN and BDNF-eMSC + iMN groups than in the BDNF-eMSC group (Fig. 6e and h). We assessed neurite outgrowth induction by co-culturing BDNF-eMSC and iMN in 3D spheroid platforms. We attached the BDNF-eMSC and iMN 3D spheroids to laminin-coated plates for 10 days and assessed neurite outgrowth from the 3D spheroids on day 10 using a neurite outgrowth assay kit. In the red fluorescence staining and intensity analysis, neurite outgrowth was significantly higher in the BDNF-eMSC + iMN group than in the BDNF-eMSC and iMN groups. Furthermore, neurite outgrowth was significantly higher in the iMN group than in the BDNF-eMSC group (Fig. 6f and h). Full-length western blot images are presented in Additional file 4: Fig. 4.

### BDNF-eMSC and iMN supported neural circuitry and connection in 3D co-culture spheroid in vitro

**Fig. 7:**
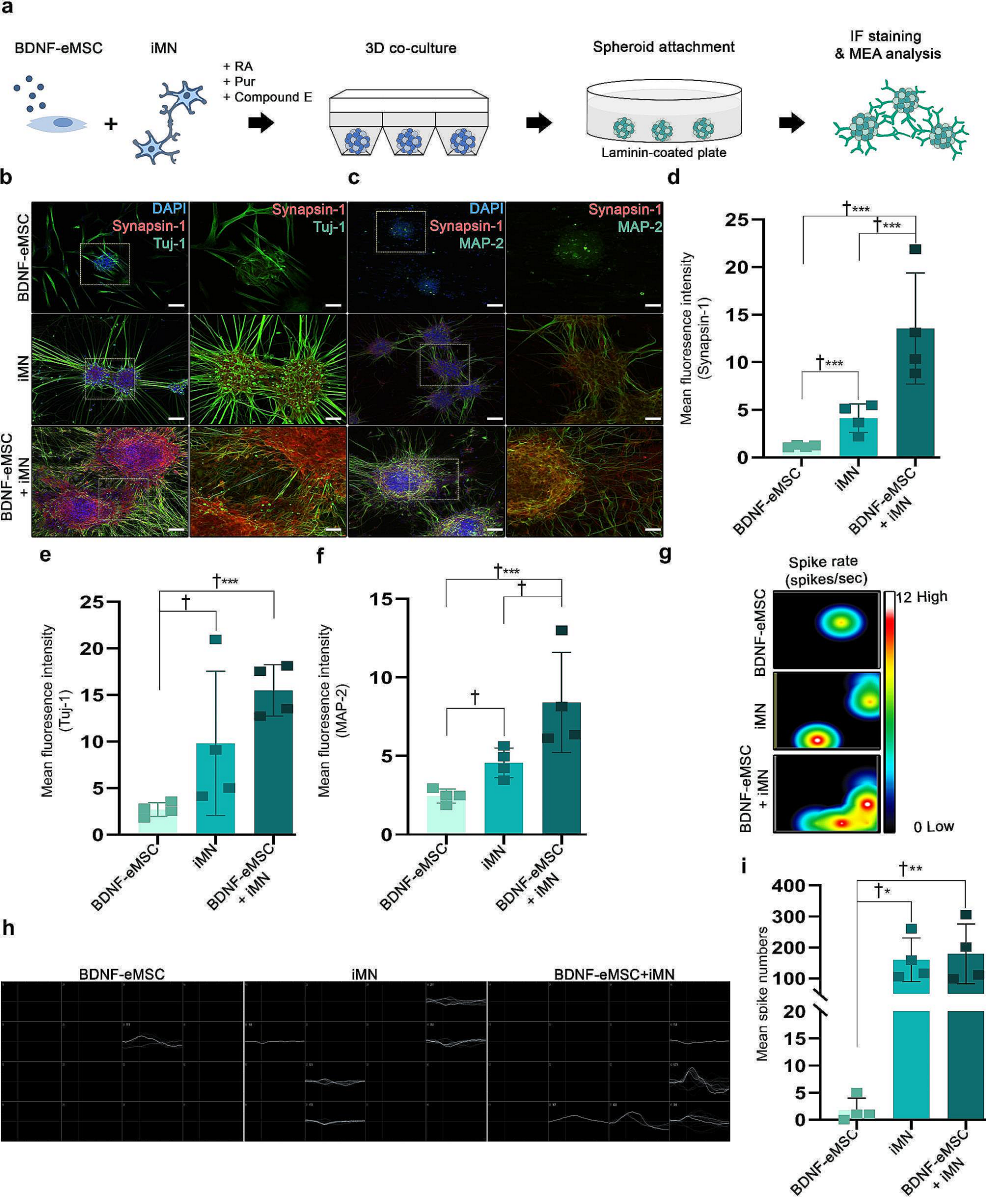
Synergistic effect of promoting synaptic connections and neural networks by BDNF-eMSC and iMN 3D co-culture platform during the differentiation of mature motor neurons. (**a**) Schematic of the assessments of BDNF-eMSC and iMN 3D co-culture in promoting the differentiation of motor neurons, synaptic connections, and neural networking using IF staining and MEA analysis in vitro assay. (**b**) Representative IF images of synapsin-1 and Tuj-1 in 3D co-culture on day 10. (**c**) Representative IF images of synapsin-1 and MAP-2 in 3D co-culture on day 10. (**d**) Quantification of the fluorescence intensity of synapsin-1 in 3D co-culture on day 10. (**e**) Quantification of the fluorescence intensity of Tuj-1 in 3D co-culture on day 10. (**f**) Quantification of the fluorescence intensity of MAP-2 in 3D co-culture on day 10. (**g**) Representative images of heatmap activity for plate-wide visualization of spike or beat rates and amplitudes on MEAs (3D BDNF-eMSC, *n* = 4; 3D iMN, *n* = 4 and 3D BDNF-eMSC + iMN, *n* = 4). (**h**) Measurement of active electrodes per well. **i** Measure of average number of spikes of active electrodes per well (3D BDNF-eMSC, *n* = 4; 3D iMN, *n* = 4 and 3D BDNF-eMSC + iMN, *n* = 4). Data are presented as mean ± SEM. Statistical significance was estimated using the Kruskal–Wallis test with *post hoc* analysis and the Mann–Whitney (†) test with the least significant difference *post hoc* analysis (*); *, † *P* < 0.05, **†† *p* < 0.01, *** *p* < 0.001. Scale bars = 50 μm. BDNF-eMSC, BDNF over-expressing engineered mesenchymal stem cells; iMN, induced pluripotent stem cell-derived motor neuron; IF, Immunofluorescence staining; MEA, multi-electrode arrays

We showed that cell transplantation using the combination of BDNF-eMSC and iMN increased the differentiation of mature motor neurons and growth density of the neuronal process in injured SC; however, it was difficult to confirm the possible mechanism in vivo. We hypothesized that the 3D co-culture of BDNF-eMSC and iMN would increase the neural circuitry and connection during MN differentiation and maturation. To confirm this hypothesis, we analyzed the induction of neural circuitry and connection by co-culturing BDNF-eMSC and iMN on 3D spheroid platforms during the differentiation of mature iMN. Synaptic connections and local neural networks were assessed in the 3D co-culture using synapsin-1, Tuj-1, MAP-2 markers, and MEA analysis on day 10 (Fig. 7a). The IF staining showed that synapsin-1, Tuj-1, and MAP-2 expression were higher in the BDNF-eMSC + iMN group than in the BDNF-eMSC and iMN groups (Fig. 7b - f). Dendrite connections between spheroids were confirmed in the iMN and BDNF-eMSC + iMN groups, but not in the BDNF-eMSC group (Fig. 7b and c). The IF staining confirmed that the fluorescence intensity of synapsin-1, Tuj-1, and MAP-2 in the iMN and BDNF-eMSC + iMN groups was significantly higher than that in the BDNF-eMSC group (Fig. 7d-f). Electrophysiology of the 3D spheroid during mature MN differentiation was confirmed using MEA. Neural spikes and activities increased in the iMN and BDNF-eMSC + iMN groups than in the BDNF-eMSC group. MEA showed a higher number of spikes in the iMN and BDNF-eMSC + iMN groups than in the BDNF-eMSC group (Fig. 7g-i). Taken together, we confirmed the successful neural circuitry and connection during the maturation of MN generated from the 3D co-culture platform.

## Discussion

Current pharmacological or physical rehabilitation-based therapies for chronic SCI are limited and primarily focus on managing symptoms such as pain or muscle stiffness. Moreover, treatments that can clinically improve motor or sensory function in patients with chronic SCI trauma are lacking [31–35]. Clinical therapeutic trials for overcoming SCI include neuronal protection and regeneration approaches. Neuroregenerative trials have been used to enhance exogenous supplement using various stem cells in chronic SCI model. These stem cells include Schwann cells, OECs, MSCs, neural stem/progenitor cells, ESCs, and iPSC-derived cells [3, 8]. Trials aimed at identifying the ideal cell types and transplantation strategies in chronic SCI are required to achieve effective stem cell transplantation in SCI. In this study, we aimed to investigate the combination transplant of BDNF-eMSC and iMNP in a chronic SCI model.

MSC cell transplantation in chronic SCI offers promising neuron regenerative strategies. Previous studies have reported significant clinical improvement with MSC transplantation in a chronic SCI model [16]. However, the efficacy of cell engraftment is low owing to the distinct pathology of chronic SCI. Therefore, modulation of the microenvironment in chronic SCI is required to enhance the efficacy of transplantation [30]. Based on previous studies, we aimed to increase the efficacy of cell engraftment and differentiation at the lesion site using BDNF-eMSCs in a chronic SCI model. Previous studies have reported the neuroprotective therapeutic effects of BDNF-eMSC in neonatal hypoxic-ischemic, traumatic brain injury, and neurogenic bladder models in rats [17, 18, 25, 30]. However, to therapeutic efficacy, safety is another critical issue for successful clinical translation. BDNF-eMSC was established using a lentiviral vector encoding the c-Myc, the reprogramming factor, tumorigenicity is a major concern for their in vivo. Previous studies findings suggest that the BDNF-eMSC is safety and ready to use and therapeutic efficacy confirmed in neonatal hypoxic-ischemic, traumatic brain injury model in rats and cardiac repair [17, 18, 24]. We performed an in vitro assay to assess BDNF expression in BDNF-eMSC and naïve MSC before cell transplantation. Our data suggest that BDNF-eMSC effectively increased BDNF expression more than naïve MSC (Fig. 1c - f). After cell seeding, BDNF expression decreased on day 7 compared with day 2, but continued BDNF expression was confirmed (Fig. 1d - g).

Recovery of motor function in chronic SCI model is still limited. Therefore, a combination treatment strategy involving various approaches must be considered to improve motor functions in chronic SCI models [36]. We attempted a combination cell transplantation strategy involving BDNF-eMSC and iMNP to increase the survival of engrafted cells at the lesion site and enhance the differentiation capacity of motor neurons in a chronic SCI model. We used iMNP cells to promote motor neuron differentiation at the lesion site. We generated iPSC-derived motor neurons using a previously reported small molecule approach [28, 29]. Our results suggest that iPSC-derived motor neurons were successfully differentiated in vitro (Fig. 2a-c), and the iMNP cell phenotype was confirmed before cell transplantation using the OLIG2 marker (Fig. 2d). We performed combination cell transplantation of irradiated BDNF-eMSC and iMNP in a contusive chronic SCI model. At 6 weeks post-injury, BDNF-eMSC + iMNP group containing both types of cells (1:1) in 10 µL PBS was transplanted to the lesion site. Interestingly, BDNF-eMSC and iMNP combination cell transplantation improved clinical recovery and incidence rate than PBS and single-cell transplantation groups in the chronic SCI model (Fig. 3a and b). At 12 weeks post-injury, we discovered that transplanted iMNP in the iMNP and BDNF-eMSC + iMNP groups remained at the lesion site, but no BDNF-eMSC were detected (Fig. 3c). Previous studies have reported that irradiated cultured HGF (Hepatocyte growth factor) over-expressing engineered mesenchymal stem cell (HGF-eMSC) exhibited decreased proliferation rates in vitro culture and no tumor was detected in in vivo tumorigenicity testing using nude mice [24]. Our result revealed that irradiated the culture BDNF-eMSC exhibited decreased BDNF expression on day 7 (Fig. 1g), and at 6 weeks after implantation, most of the BDNF-eMSC did not remain at the lesion site using microscopic observations (Fig. 3c). These results suggest that genetically engineered cell is a suitable combination cell transplantation strategy in chronic SCI models.

Previous studies have reported that allogenic bone marrow-derived MSC transplantation without cell manipulation in acute and chronic SCI mainly resulted in astrocytic differentiation at the lesion site [16, 37, 38]. Our research discovered that BDNF-eMSC transplantation in chronic SCI increased the oligodendrocyte and neuron cells compared with PBS. However, the BDNF-eMSC group showed less neuron differentiation at the lesion site than the iMNP group (Fig. 5a - f). Another study reported that human iMNP cell transplantation in an acute SCI model resulted in transplanted human iMNP with a motor neuron lineage of mixed maturation state in the ventral horns [23]. Our research observed higher SMI-32 expression in transplanted iMNP cell in the iMNP and BDNF-eMSC + iMNP groups than that in the PBS and BDNF-eMSC groups at 12 weeks post-injury (Fig. 4a and b). Our results suggest that iMNP directly influences mature motor neuron differentiation more than BDNF-eMSC at the lesion site. Transplanted hMNP cell increased endogenous neuronal survival and promote neurite branching [23]. We confirmed that BDNF-eMSC and iMNP combination cell transplantation increased axonal regeneration, as indicated by MAP-2 expression, compared with PBS. The BDNF-eMSC and iMNP groups showed significantly higher MAP-2 expression than the iMNP group at the lesion site (Fig. 4c and d). Our results suggest that mature motor neuron differentiation and growth density of neuronal processes are enhanced by the synergistic effects of BDNF-eMSC and iMNP combination cell transplantation at the lesion site in the chronic SCI model (Figs. 3b and 4b and d). In this study, we were able to confirm that transplanted cells at the lesion site could promote the growth density of neuronal processes using MAP-2 and GAP-43 markers. However, the limitation is that we could not detect axonal regeneration using retrovirus or anterograde tracer BDA. In future studies, axonal regeneration detection using anterograde tracer BDA is needed at the lesion site after cell transplantation.

Other studies have reported that motor neurons respond to neurotrophic cues and express and secret growth factors. Moreover, hMNPs express and secrete neurotrophic factors that promote axonal growth and protect neurons from cell death [22, 23, 39]. We confirmed that mature motor neuron differentiation and BDNF expression were increased at the lesion site by BDNF-eMSC + iMNP combination cell transplantation in chronic SCI (Figs. 3h and 4a and b). In addition, axonal regeneration was promoted at the lesion site (Fig. 4d). However, it was complicated to confirm the possible mechanism in vivo. We hypothesized that the BDNF-eMSC and iMN might synergically promote neurite outgrowth induction during motor neuron differentiation and maturation through BDNF expression. We co-cultured BDNF-eMSC and iMN in 2D and 3D spheroid platforms during mature motor neuron differentiation and assessed the neurite outgrowth in vitro assay to confirm this hypothesis. As in previous studies, the BDNF-eMSC + iMN group had significantly higher mature motor neuron differences and BDNF expression than the iMN group. In addition, neurite outgrowth was significantly promoted in the BDNF-eMSC + iMN group (Fig. 6f and h). However, BDNF-eMSC has a paracrine effect on motor neuron differentiation and neurite outgrowth promotion (Fig. 6f, g and h). Our results suggest that BDNF-eMSC and iMN co-cultures play an essential role in promoting mature motor neuron differentiation and neurite outgrowth. Additionally, the in vitro assay confirmed that the co-culture of BDNF-eMSC and iMN could promote functional synaptic connections and neural networks during the differentiation of mature motor neurons. These results show successful neural circuitry and connection during maturation of MN generated from the 3D co-culture platform. We were able to confirm that the synergistic effect of BDNF-eMSC + iMN promoted the differentiation of mature motor neurons and neural networks in vitro (Fig. 7). However, in future studies, it is necessary to confirm that the transplanted cells at the lesion site promote functional synaptic connections and local neural networks after transplantation of a combination of BDNF-eMSCand iMNP cells.

In summary, this study confirms that behavioral abilities were recovered through the induction of differentiation of mature motor neurons at the lesion site by transplanting a combination of BDNF-eMSC and iMNP cells in a chronic SCI model, suggesting the therapeutic efficacy of the transplantation strategy using a combination of genetically engineered cells and iPSCs in a chronic SCI rat model. However, the limitation of this study is the lack of explanation of the mechanisms supporting the synergistic effect of transplantation of combined genetically engineered cells and iPSCs in the chronic SCI model. In future studies, it will be necessary to confirm the mechanisms of the synergistic effects of transplantation of a combination of cell types using RNA sequencing (RNA-seq) or single-cell analysis at the lesion site. In addition, it is necessary to study the effect of neural regeneration by the differentiation of motor neurons and BDNF expression according to cell ratio and number of transplants. To reduce variation in animal experiments, a sample size sufficient for statistical analysis should be calculated using a few free software packages (G power, power sample).

## Conclusion

To our knowledge, this study demonstrates that the combination cell transplantation of BDNF-eMSC and iMNP improves behavioral recovery in the chronic SCI model. At 12 weeks post-injury, the transplanted iMNP predominantly differentiated into mature motor neurons. The BDNF-eMSC exerted a paracrine effect on neuron regeneration, as evidenced by BDNF expression at the lesion site. In vivo and in vitro, the co-culture of BDNF-eMSC and iMNP played a crucial role in motor neuron maturation and axonal regeneration through BDNF expression. Overall, our findings provide proof of concept that stem cell-based gene therapy and combination cell transplantation can enhance motor neuron maturation and BDNF expression in chronic SCI.

### Electronic supplementary material

Below is the link to the electronic supplementary material.

Supplementary Material 1

Supplementary Material 2

Supplementary Movie 1: BBB locomotor scales to evaluate the clinical recovery of behavior for 12 weeks post injury in PBS group (separate Movie file)

Supplementary Movie 2: BBB locomotor scales to evaluate the clinical recovery of behavior for 12 weeks post injury in BDNF eMSC group (separate Movie file)

Supplementary Movie 3: BBB locomotor scales to evaluate the clinical recovery of behavior for 12 weeks post injury in iMNP group (separate Movie file)

Supplementary Movie 4: BBB locomotor scales to evaluate the clinical recovery of behavior for 12 weeks post injury in BDNF eMSC+iMNP group (separate Movie file)

## Data Availability

All datasets of this article are included within the article.

## Abbreviations

AVMA: American veterinary medical association
ARRIVE: Animal research: reporting of in vivo experiments
BBB: Blood-Brain-Barrier
BBB: Basso–Beattie–Bresnahan
BSA: Bovine serum albumin
BDNF: Brain-derived neurotrophic factor
CBMCs: Cord blood mononuclear cells
DMEM: Dulbecco’s modified eagle’s medium
DMH1: Dorsomorphin homologue 1
DAPI: 4′,6-diamidino-2-phenylindole
eMSC: Engineered mesenchymal stem cells
ESC: Embryonic stem cell
hMSC: Human mesenchymal stem cells
HGF: Hepatocyte growth factor
iPSC: Induced pluripotent stem cell
iNEP: Induced pluripotent stem cell derived Neuro epithelial progenitor
iMNP: Induced pluripotent stem cell derived motor neuron progenitor cells
iMN: Induced pluripotent stem cell derived motor neuron
iMature MNs: Induced pluripotent stem cell derived mature motor neurons
IL: Intra lesional
IF: Immuno Fluorescence
MNs: Motor neuron cells
MASCIS: Multicenter animal spinal cord injury study
MAP-2: Microtubule-Associated protein-2
NSPCs: Neural stem and progenitor cells
OECs: Olfactory ensheathing cells
OCT: Optimal cutting temperature
PBS: Phosphate-buffered saline
PFA: Paraformaldehyde
PMSF: Phenyl methyl sulfonyl fluoride
Pur: Pumorphamine
RT: Room temperature
RA: Retinoic acid
SCI: Spinal cord injury
SC: Spinal cord
SD: Sprague–Dawley
tTA: Tetra cycline trans activator
TBS: Tris-buffered saline
TBST: Tris-buffered saline with 0.05% Tween-20
TSCI: Traumatic spinal cord injury
WB: Western blot

## References

[1] Quadri SA, Farooqui M, Ikram A, Zafar A, Khan MA, Suriya SS, Claus CF, Fiani B, Rahman M, Ramachandran A (2020). Recent update on basic mechanisms of spinal cord injury. Neurosurg Rev.

[2] Barbiellini Amidei C, Salmaso L, Bellio S, Saia M (2022). Epidemiology of traumatic spinal cord injury: a large population-based study. Spinal Cord.

[3] Kim YH, Ha KY, Kim SI (2017). Spinal cord Injury and related clinical trials. Clin Orthop Surg.

[4] Pang QM, Chen SY, Xu QJ, Fu SP, Yang YC, Zou WH, Zhang M, Liu J, Wan WH, Peng JC, Zhang T (2021). Neuroinflammation and Scarring after spinal cord Injury: therapeutic roles of MSCs on inflammation and glial scar. Front Immunol.

[5] Al Mamun A, Monalisa I, Tul Kubra K, Akter A, Akter J, Sarker T, Munir F, Wu Y, Jia C, Afrin Taniya M, Xiao J (2021). Advances in immunotherapy for the treatment of spinal cord injury. Immunobiology.

[6] Eli I, Lerner DP, Ghogawala Z (2021). Acute traumatic spinal cord Injury. Neurol Clin.

[7] Fischer I, Dulin JN, Lane MA (2020). Transplanting neural progenitor cells to restore connectivity after spinal cord injury. Nat Rev Neurosci.

[8] Assinck P, Duncan GJ, Hilton BJ, Plemel JR, Tetzlaff W (2017). Cell transplantation therapy for spinal cord injury. Nat Neurosci.

[9] Tetzlaff W, Okon EB, Karimi-Abdolrezaee S, Hill CE, Sparling JS, Plemel JR, Plunet WT, Tsai EC, Baptiste D, Smithson LJ (2011). A systematic review of cellular transplantation therapies for spinal cord injury. J Neurotrauma.

[10] Lu P, Woodruff G, Wang Y, Graham L, Hunt M, Wu D, Boehle E, Ahmad R, Poplawski G, Brock J (2014). Long-distance axonal growth from human induced pluripotent stem cells after spinal cord injury. Neuron.

[11] Yang N, Zuchero JB, Ahlenius H, Marro S, Ng YH, Vierbuchen T, Hawkins JS, Geissler R, Barres BA, Wernig M (2013). Generation of oligodendroglial cells by direct lineage conversion. Nat Biotechnol.

[12] Takahashi K, Yamanaka S (2006). Induction of pluripotent stem cells from mouse embryonic and adult fibroblast cultures by defined factors. Cell.

[13] Kramer AS, Harvey AR, Plant GW, Hodgetts SI (2013). Systematic review of induced pluripotent stem cell technology as a potential clinical therapy for spinal cord injury. Cell Transpl.

[14] Sun L, Wang F, Chen H, Liu D, Qu T, Li X, Xu D, Liu F, Yin Z, Chen Y (2019). Co-transplantation of Human umbilical cord mesenchymal stem cells and human neural stem cells improves the outcome in rats with spinal cord Injury. Cell Transpl.

[15] Siddiqui AM, Khazaei M, Fehlings MG (2015). Translating mechanisms of neuroprotection, regeneration, and repair to treatment of spinal cord injury. Prog Brain Res.

[16] Kim JW, Ha KY, Molon JN, Kim YH (2013). Bone marrow-derived mesenchymal stem cell transplantation for chronic spinal cord injury in rats: comparative study between intralesional and intravenous transplantation. Spine (Phila Pa 1976).

[17] Ahn SY, Sung DK, Chang YS, Sung SI, Kim YE, Kim HJ, Lee SM, Park WS. BDNF-Overexpressing Engineered mesenchymal stem cells enhances their therapeutic efficacy against severe neonatal hypoxic ischemic brain Injury. Int J Mol Sci 2021, 22. 10.3390/ijms222111395 PMC8583727 34768827

[18] Choi BY, Hong DK, Kang BS, Lee SH, Choi S, Kim HJ, Lee SM, Suh SW. Engineered Mesenchymal stem cells over-expressing BDNF protect the Brain from Traumatic Brain Injury-Induced neuronal death, neurological deficits, and cognitive impairments. Pharmaceuticals (Basel) 2023, 16. 10.3390/ph16030436 PMC10054459 36986535

[19] Ahn SY, Sung DK, Kim YE, Sung S, Chang YS, Park WS (2021). Brain-derived neurotropic factor mediates neuroprotection of mesenchymal stem cell-derived extracellular vesicles against severe intraventricular hemorrhage in newborn rats. Stem Cells Transl Med.

[20] Khazaei M, Siddiqui AM, Fehlings MG (2014). The potential for iPS-Derived stem cells as a therapeutic strategy for spinal cord Injury: opportunities and challenges. J Clin Med.

[21] Nogradi A, Pajer K, Marton G (2011). The role of embryonic motoneuron transplants to restore the lost motor function of the injured spinal cord. Ann Anat.

[22] Lukovic D, Valdes-Sanchez L, Sanchez-Vera I, Moreno-Manzano V, Stojkovic M, Bhattacharya SS, Erceg S (2014). Brief report: astrogliosis promotes functional recovery of completely transected spinal cord following transplantation of hESC-derived oligodendrocyte and motoneuron progenitors. Stem Cells.

[23] Rossi SL, Nistor G, Wyatt T, Yin HZ, Poole AJ, Weiss JH, Gardener MJ, Dijkstra S, Fischer DF, Keirstead HS (2010). Histological and functional benefit following transplantation of motor neuron progenitors to the injured rat spinal cord. PLoS ONE.

[24] Park BW, Jung SH, Das S, Lee SM, Park JH, Kim H, Hwang JW, Lee S, Kim HJ, Kim HY (2020). In vivo priming of human mesenchymal stem cells with hepatocyte growth factor-engineered mesenchymal stem cells promotes therapeutic potential for cardiac repair. Sci Adv.

[25] Tian WJ, Jeon SH, Zhu GQ, Kwon EB, Kim GE, Bae WJ, Cho HJ, Ha US, Hong SH, Lee JY (2021). Effect of high-BDNF microenvironment stem cells therapy on neurogenic bladder model in rats. Transl Androl Urol.

[26] Rim YA, Nam Y, Ju JH (2019). Application of Cord Blood and Cord Blood-Derived Induced Pluripotent Stem cells for cartilage regeneration. Cell Transpl.

[27] Nam Y, Rim YA, Jung SM, Ju JH (2017). Cord blood cell-derived iPSCs as a new candidate for chondrogenic differentiation and cartilage regeneration. Stem Cell Res Ther.

[28] Du ZW, Chen H, Liu H, Lu J, Qian K, Huang CL, Zhong X, Fan F, Zhang SC (2015). Generation and expansion of highly pure motor neuron progenitors from human pluripotent stem cells. Nat Commun.

[29] Li XJ, Du ZW, Zarnowska ED, Pankratz M, Hansen LO, Pearce RA, Zhang SC (2005). Specification of motoneurons from human embryonic stem cells. Nat Biotechnol.

[30] Lee JY, Ha KY, Kim JW, Seo JY, Kim YH (2014). Does extracorporeal shock wave introduce alteration of microenvironment in cell therapy for chronic spinal cord injury?. Spine (Phila Pa 1976).

[31] Curtis E, Martin JR, Gabel B, Sidhu N, Rzesiewicz TK, Mandeville R, Van Gorp S, Leerink M, Tadokoro T, Marsala S (2018). A first-in-Human, phase I study of neural stem cell transplantation for chronic spinal cord Injury. Cell Stem Cell.

[32] Saulino M, Averna JF (2016). Evaluation and management of SCI-Associated Pain. Curr Pain Headache Rep.

[33] Gwak YS, Kim HY, Lee BH, Yang CH (2016). Combined approaches for the relief of spinal cord injury-induced neuropathic pain. Complement Ther Med.

[34] McIntyre A, Mays R, Mehta S, Janzen S, Townson A, Hsieh J, Wolfe D, Teasell R (2014). Examining the effectiveness of intrathecal baclofen on spasticity in individuals with chronic spinal cord injury: a systematic review. J Spinal Cord Med.

[35] Emamhadi M, Alijani B, Andalib S (2016). Long-term clinical outcomes of spinal accessory nerve transfer to the suprascapular nerve in patients with brachial plexus palsy. Acta Neurochir (Wien).

[36] Gomes-Osman J, Cortes M, Guest J, Pascual-Leone A (2016). A systematic review of experimental strategies aimed at improving motor function after Acute and chronic spinal cord Injury. J Neurotrauma.

[37] Kim YC, Kim YH, Kim JW, Ha KY (2016). Transplantation of mesenchymal stem cells for Acute spinal cord Injury in rats: comparative study between Intralesional Injection and Scaffold based transplantation. J Korean Med Sci.

[38] Kang ES, Ha KY, Kim YH (2012). Fate of transplanted bone marrow derived mesenchymal stem cells following spinal cord injury in rats by transplantation routes. J Korean Med Sci.

[39] Erceg S, Ronaghi M, Oria M, Rosello MG, Arago MA, Lopez MG, Radojevic I, Moreno-Manzano V, Rodriguez-Jimenez FJ, Bhattacharya SS (2010). Transplanted oligodendrocytes and motoneuron progenitors generated from human embryonic stem cells promote locomotor recovery after spinal cord transection. Stem Cells.

