# Supplementary material for: Combination of induced pluripotent stem cell-derived motor neuron progenitor cells with irradiated brain-derived neurotrophic factor over-expressing engineered mesenchymal stem cells enhanced restoration of axonal regeneration in a chronic spinal cord injury rat model
Source: Stem Cell Res Ther. 2024 Jun 18;15:173. doi: 10.1186/s13287-024-03770-9 (PMC11184802; doi:10.1186/s13287-024-03770-9)
Supplement: Supplementary file 1 — Supplementary Material 1 [file 13287_2024_3770_MOESM1_ESM.docx]

**
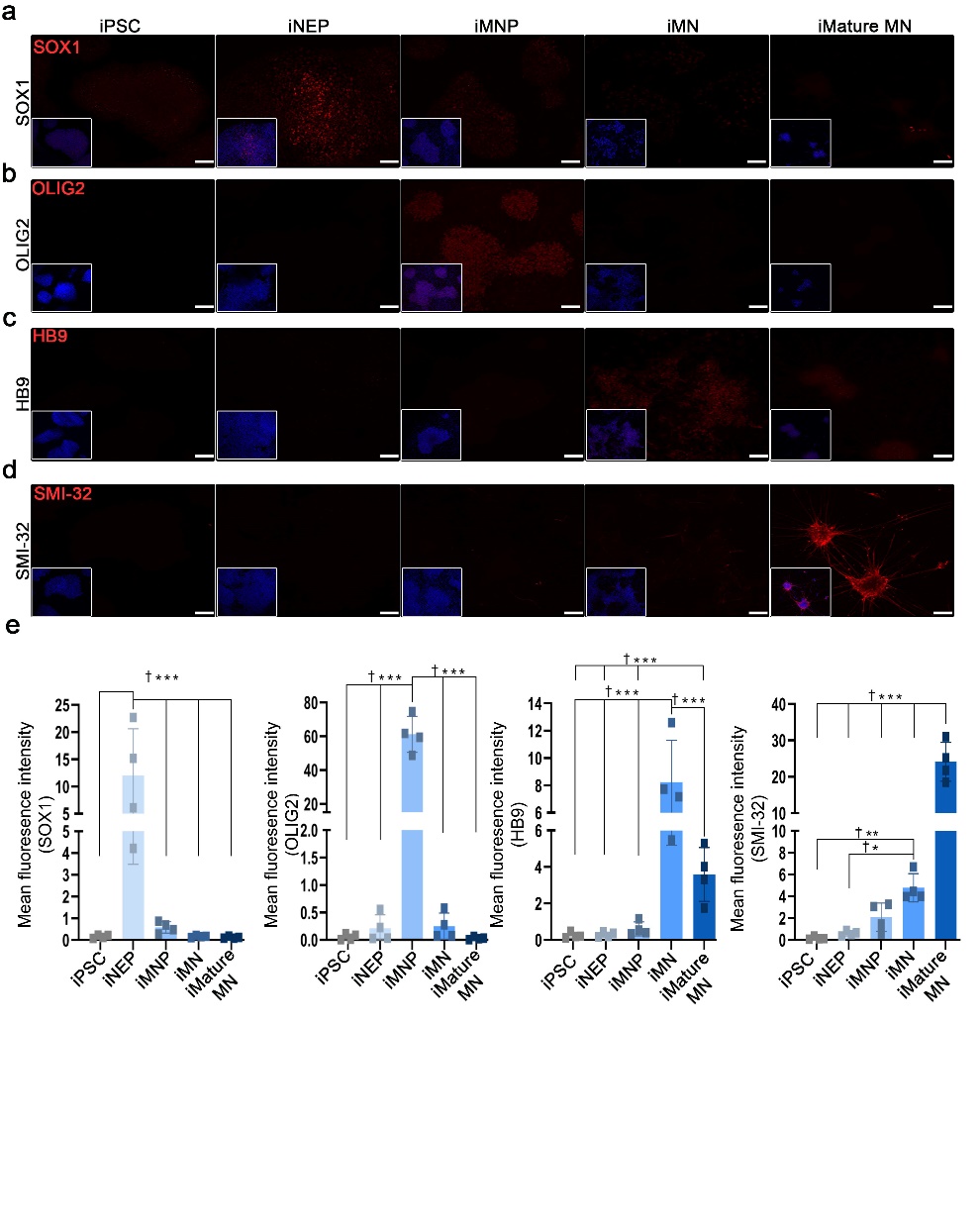
**

**Additional file 1: Figure S1. Generation of induced pluripotent stem cell (iPSC)-derived motor neurons.**

**a** Representative fluorescence time course images of iPSC, iNEP, iMNP, iMN, and iMature MN using SOX1 markers in the course of time. **b** Representative fluorescence images of iPSC, iNEP, iMNP, iMN, and iMature MN using OLIG2 markers in the course of time. **c** Representative fluorescence images of iPSC, iNEP, iMNP, iMN, and iMature MN using HB9 markers in the course of time. **d** Representative fluorescence images of iPSC, iNEP, iMNP, iMN, and iMature MN using SMI-32 markers in the course of time. **e** Quantification of the fluorescence intensity of markers at each stage (iNEP: n=4, iMNP; n=4, iMN: n=4, and iMature MN: n=4). Data are presented as mean ± SEM. Statistical significance was estimated using the Kruskal–Wallis test with *post hoc* analysis and the Mann–Whitney (†) test with the least significant difference *post hoc* analysis (*); *, † *P* < 0.05, **†† *p* < 0.01. Scale bars = 50 μm. iPSCs, induced pluripotent stem cell; iNEPs, induced pluripotent stem cell-derived neuron epithelial progenitor cells; iMNPs, induced pluripotent stem cell-derived motor neuron progenitor cells; iMNs, induced pluripotent stem cell-derived motor neuron cells; iMature MNs, induced pluripotent stem cell-derived mature motor neuron cells; IF, Immunofluorescence staining; SOX1 = iNEP, OLIG2 = iMNP, HB9 = iMN, SMI-32= iMature MN.

**
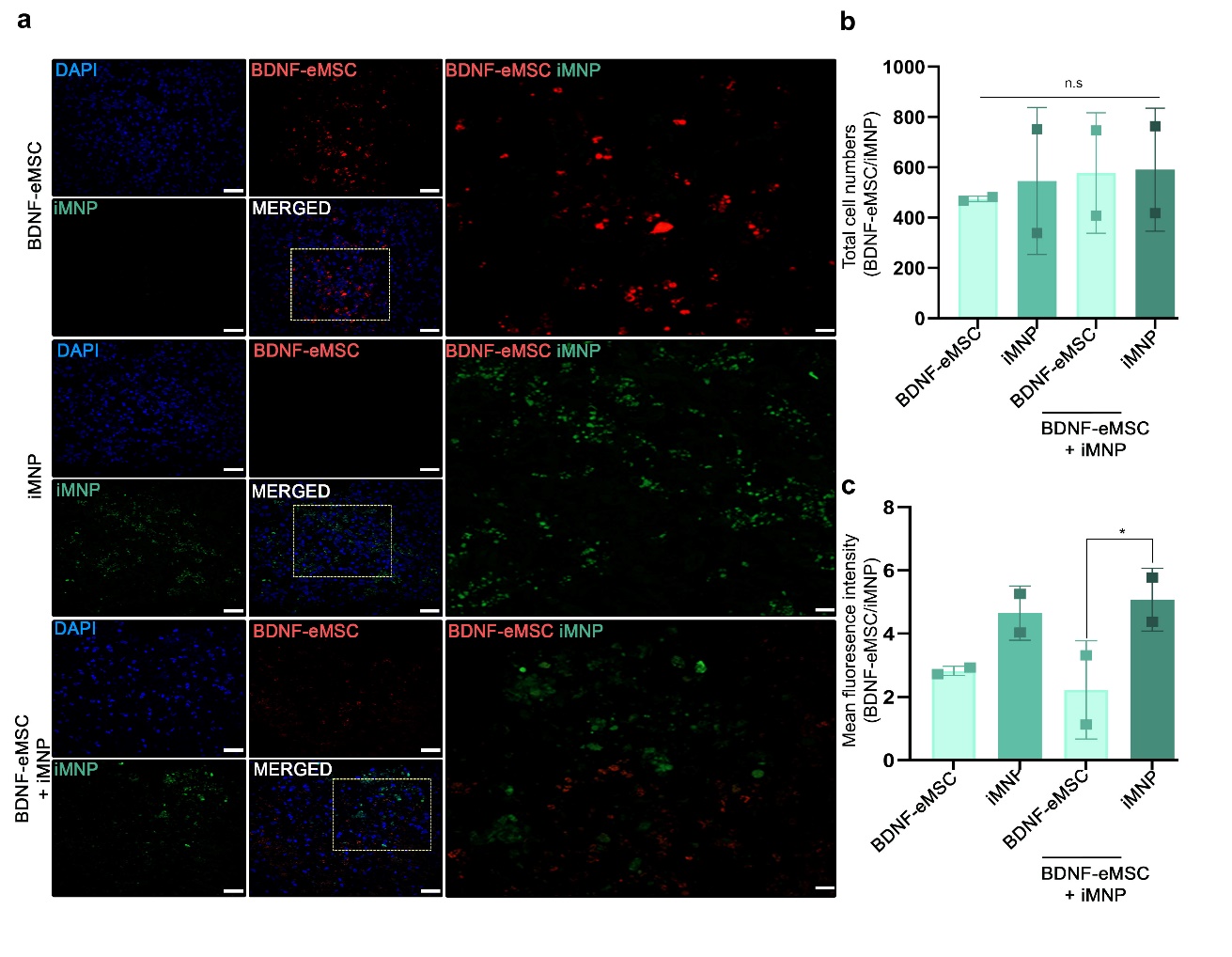
**

**Additional file 2: Figure S2. Transplanted cells at the lesion site one week after cell transplantation.**

**a** Representative fluorescence images of PKH26 (BDNF-eMSC) and PKH 67 (iMNP) at the lesion site one week post-cell transplantation. **b** Numbers of engrafted cells at the lesion site (BDNF-eMSC: n=2, iMNP; n=2, BDNF-eMSC+iMNP n=2). **c** Quantification of fluorescence intensity of engrafted cells at the lesion site (BDNF-eMSC: n=2, iMNP; n=2, BDNF-eMSC+iMNP n=2). Data are presented as mean ± SEM. Statistical significance was estimated using the Kruskal–Wallis test with *post hoc* analysis and the Mann–Whitney (†) test with the least significant difference *post hoc* analysis (*); *, † *P* < 0.05, **†† *p* < 0.01. Scale bars = 50 μm. BDNF-eMSC, Brain-derived neurotrophic factor over-expressing engineered mesenchymal stem cells; iMNPs, induced pluripotent stem cell-derived motor neuron progenitor cells; IF, Immunofluorescence staining.

**
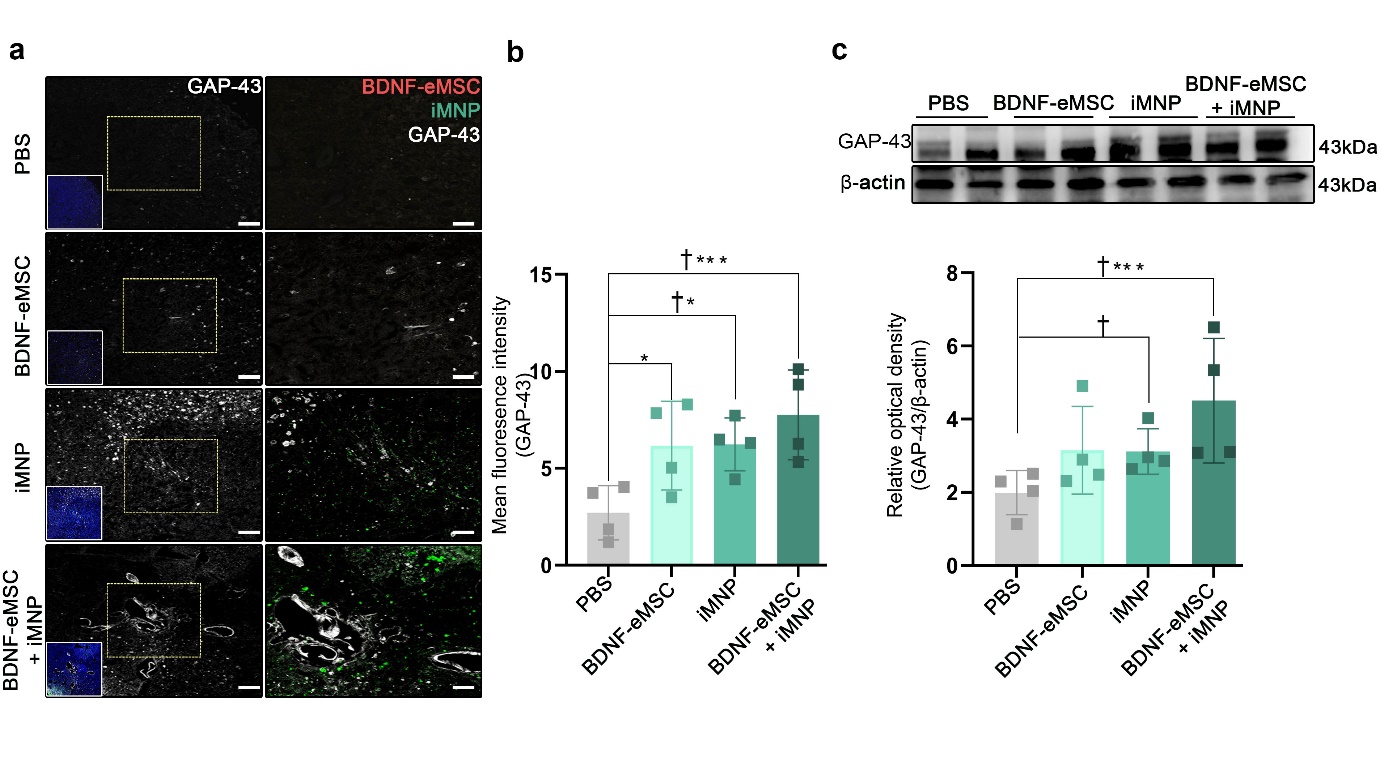
**

**Additional file 3: Figure S3. Enhancements of growth density of neuronal process by BDNF-eMSC and iMNP at the lesion site.**

**a** Representative fluorescence image of GAP-43 at the lesion site 12 weeks post-injury. **b** Quantification of fluorescence intensity of GAP-43 at the lesion site (PBS: n=4, BDNF-eMSC: n=4, iMNP: n=4, BDNF-eMSC+iMNP n=4). **c** WB results of GAP-43 expression at the lesion site segment (approximately 1 cm) (PBS: n=4, BDNF-eMSC: n=4, iMNP: n=4, BDNF-eMSC+iMNP n=4). Full-length WB images are presented in Additional file 4: Fig.4. Data are presented as mean ± SEM. Statistical significance was estimated using the Kruskal–Wallis test with *post hoc* analysis and the Mann–Whitney (†) test with the least significant difference *post hoc* analysis (*); *, † *P* < 0.05. Scale bars = 50 μm. BDNF-eMSC, Brain-derived neurotrophic factor over-expressing engineered mesenchymal stem cells; iMNP, induced pluripotent stem cell-derived motor neuron progenitor cells; IF, Immunofluorescence staining; WB, Western blot.
