# Supplementary material for: Combination of induced pluripotent stem cell-derived motor neuron progenitor cells with irradiated brain-derived neurotrophic factor over-expressing engineered mesenchymal stem cells enhanced restoration of axonal regeneration in a chronic spinal cord injury rat model
Source: Stem Cell Res Ther. 2024 Jun 18;15:173. doi: 10.1186/s13287-024-03770-9 (PMC11184802; doi:10.1186/s13287-024-03770-9)

Full-length western blot images for fig. 1f-g

a Full-length blot images for Figure 1 f and g

f. BDNF 28 and 32kDa

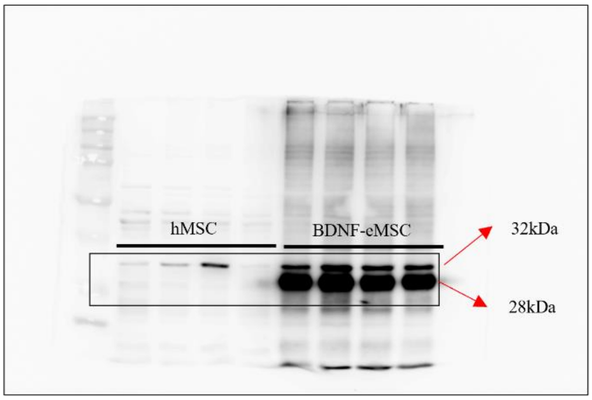

f. Beta actin 43kDa

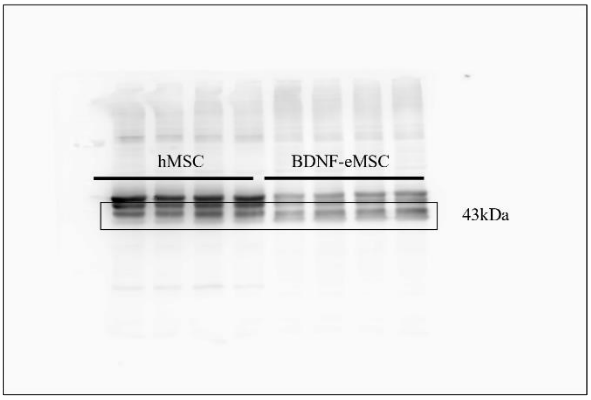

g. BDNF 28 and 32kDa

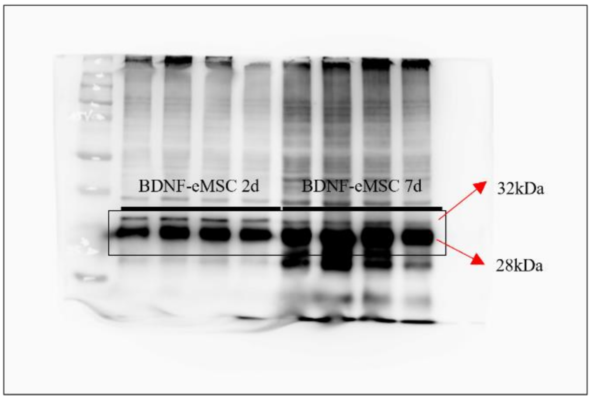

g. Beta actin 43kDa

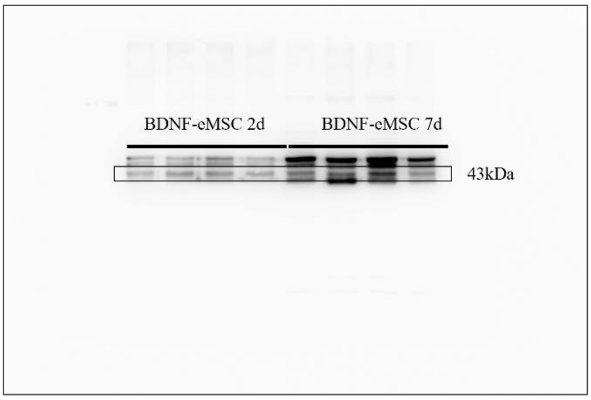

Full-length western blot images for fig. 2d

a Full-length blot images for Figure 2 d

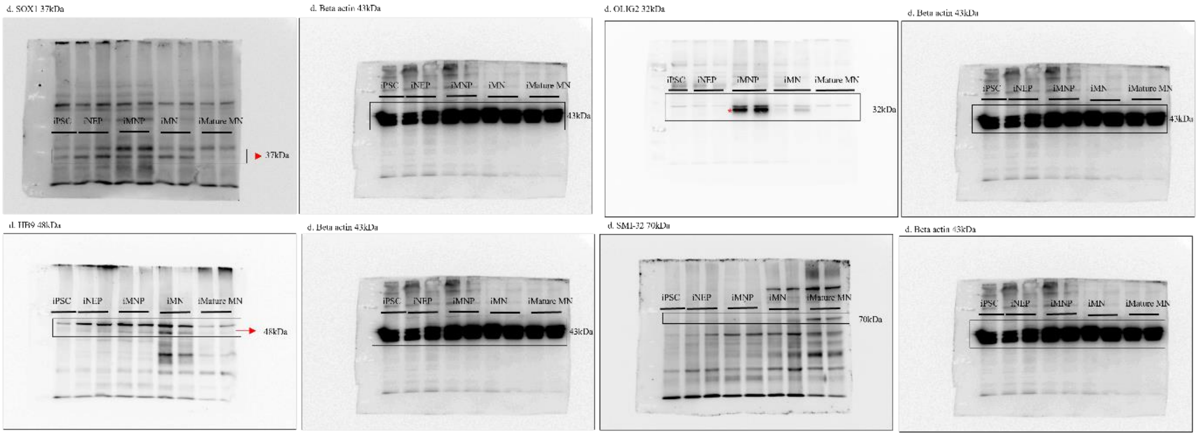

Full-length western blot images for fig. 3h

Full-length blot images for figure 3 h

h. BDNF 32kDa

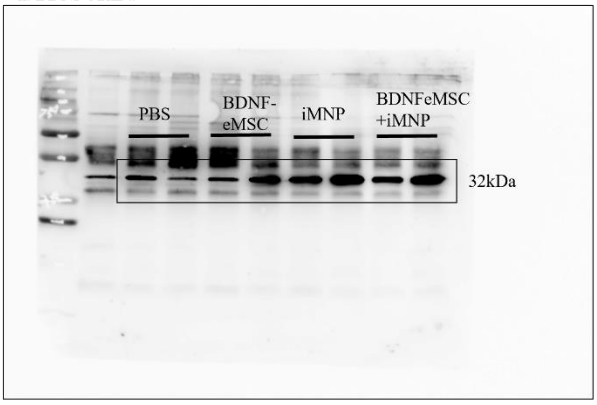

h. Beta actin 43kDa

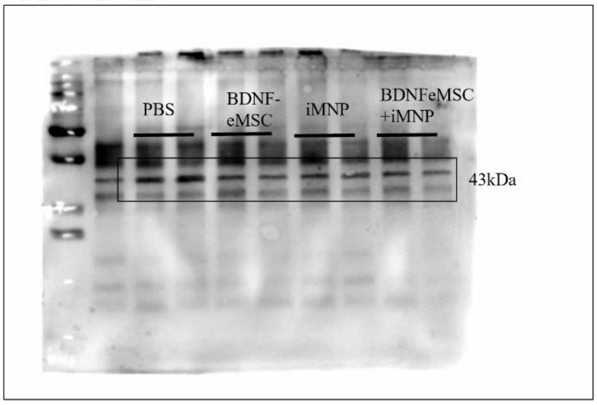

Full-length western blot images for fig. 4b and d

Full-length blot images for figure 4 b and d

b. SMI-32 145kDa

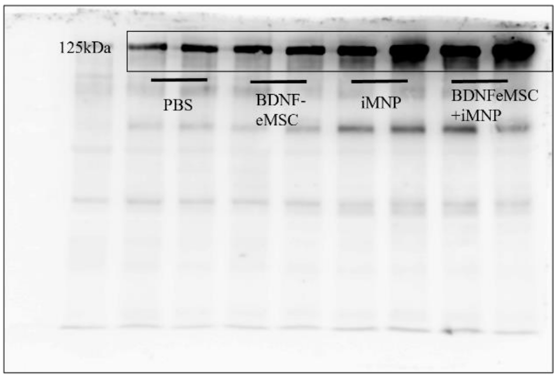

b. Beta actin 43kDa

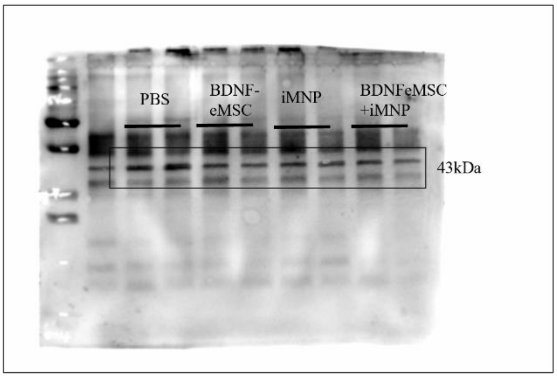

d. MAP-2 80kDa

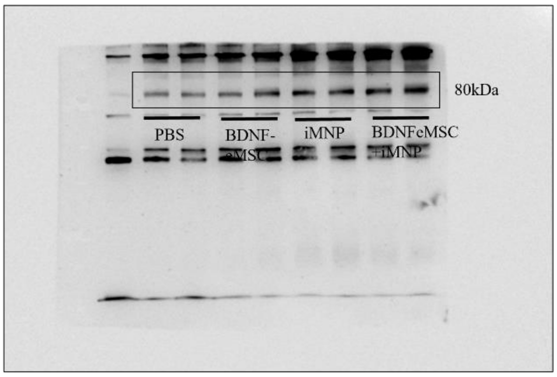

d. Beta actin 43kDa

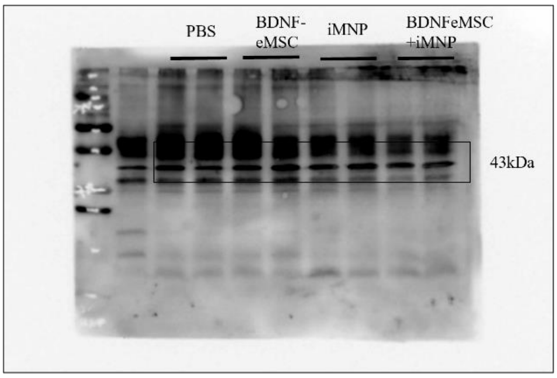

Full-length western blot images for fig. 5c and f

Full-length blot images for figure 5 c and f

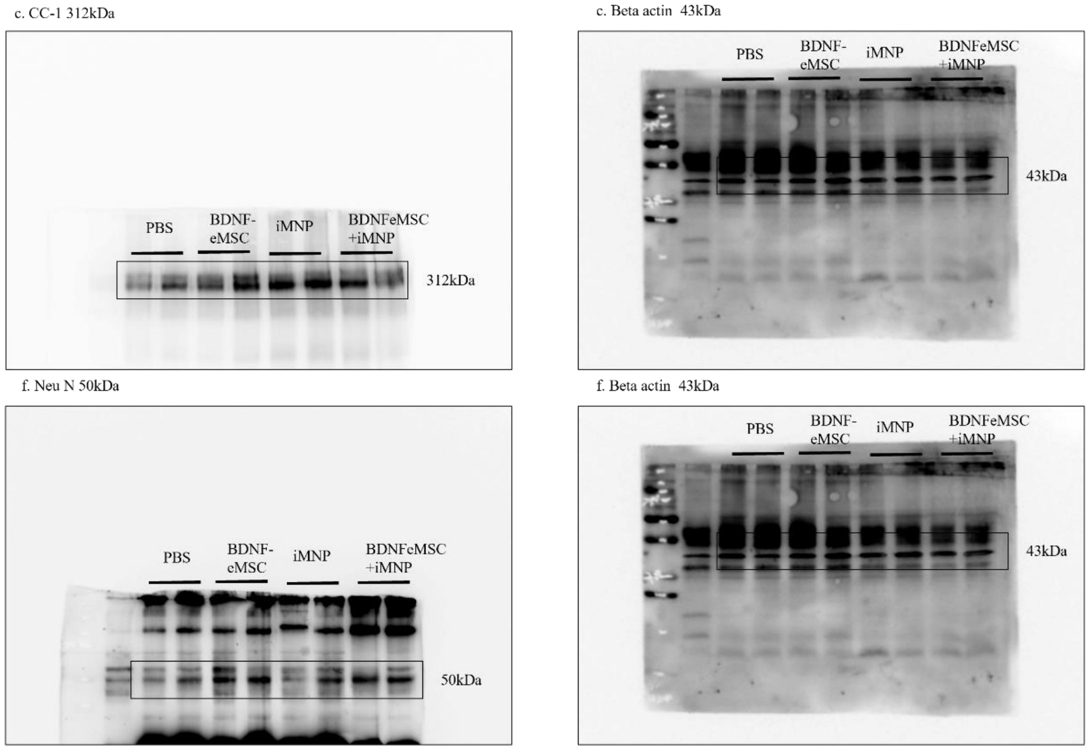

Full-length western blot images for fig. 6b

Full-length blot images for figure 6 b

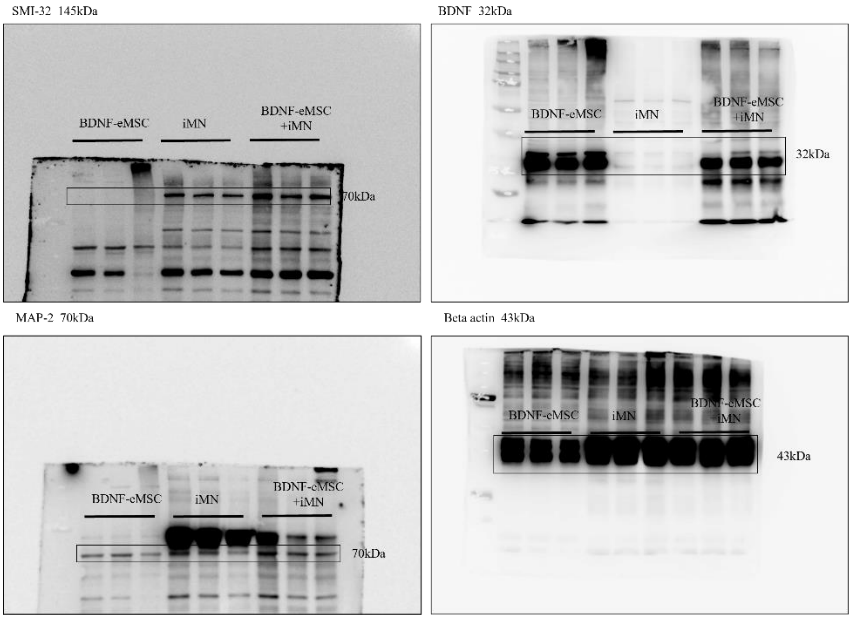

Full-length western blot images for additional Files Figure .3c

Full-length blot images for additional File Figure 3c

c. GAP-43 43kDa

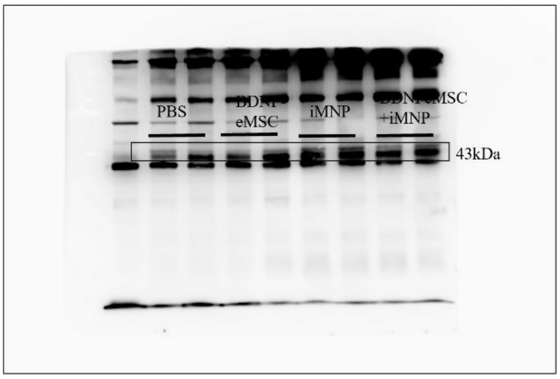

c. Beta actin 43kDa

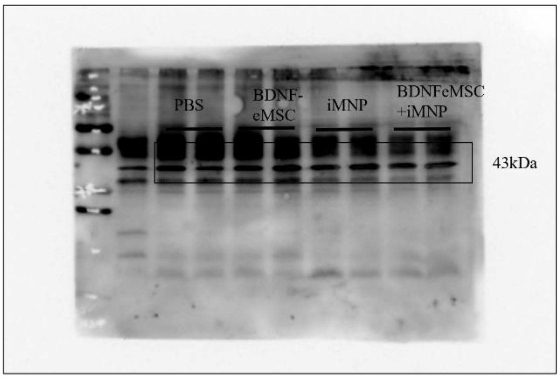

Supplement: Supplementary file 2 — Supplementary Material 2 [file 13287_2024_3770_MOESM2_ESM.pdf]
